# Genetic signs of multiple colonization events in Baltic ciscoes with radiation into sympatric spring- and autumn-spawners confined to early postglacial arrival

**DOI:** 10.1002/ece3.1299

**Published:** 2014-10-27

**Authors:** Bo Delling, Stefan Palm, Eleftheria Palkopoulou, Tore Prestegaard

**Affiliations:** 1 Department of Zoology, Swedish Museum of Natural HistoryStockholm, 10405, Sweden; 2 Department of Aquatic Resources, Institute of Freshwater Research, Swedish University of Agricultural SciencesStångholmsvägen 2, Drottningholm, 17893, Sweden; 3 Department of Bioinformatics and Genetics, Swedish Museum of Natural HistoryStockholm, 10405, Sweden; 4 Department of Zoology, Stockholm UniversityStockholm, 10405, Sweden

**Keywords:** *Coregonus*, microsatellites, mtDNA sequences, phylogeography

## Abstract

Presence of sympatric populations may reflect local diversification or secondary contact of already distinct forms. The Baltic cisco (*Coregonus albula*) normally spawns in late autumn, but in a few lakes in Northern Europe sympatric autumn and spring- or winter-spawners have been described. So far, the evolutionary relationships and taxonomic status of these main life history forms have remained largely unclear. With microsatellites and mtDNA sequences, we analyzed extant and extinct spring- and autumn-spawners from a total of 23 Swedish localities, including sympatric populations. Published sequences from Baltic ciscoes in Germany and Finland, and *Coregonus sardinella* from North America were also included together with novel mtDNA sequences from Siberian *C. sardinella*. A clear genetic structure within Sweden was found that included two population assemblages markedly differentiated at microsatellites and apparently fixed for mtDNA haplotypes from two distinct clades. All sympatric Swedish populations belonged to the same assemblage, suggesting parallel evolution of spring-spawning rather than secondary contact. The pattern observed further suggests that postglacial immigration to Northern Europe occurred from at least two different refugia. Previous results showing that mtDNA in Baltic cisco is paraphyletic with respect to North American *C. sardinella* were confirmed. However, the inclusion of Siberian *C. sardinella* revealed a more complicated pattern, as these novel haplotypes were found within one of the two main *C. albula* clades and were clearly distinct from those in North American *C. sardinella*. The evolutionary history of Northern Hemisphere ciscoes thus seems to be more complex than previously recognized.

## Introduction

A species' genetic structure represents the combined result of ongoing microevolutionary processes and historical events (e.g., Avise 2000). In Northern Europe, for example, genetic signs of postglacial recolonization from one or multiple refugia have been observed in a variety of plant and animal species (Bernatchez and Wilson 1998; Hewitt 1999). At the same time, the genetic structure of many populations in previously glaciated areas has often evolved more recently. The Holarctic genus *Coregonus* (whitefishes and ciscoes) is renowned for its elusive taxonomy and systematics, reflecting a complex mixture of past glacial events and subsequent evolution (Hudson et al. 2005). For this group, combining morphological and ecological information with molecular data has in several cases unraveled previously unknown or disputed evolutionary relationships (e.g., Turgeon and Bernatchez 2003; Østbye et al. 2006). Coregonid fishes have also served as suitable models for detailed genetic studies of rapid adaptive radiation (e.g., Mehner et al. 2010; Hudson et al. 2011).

Baltic cisco or vendace (*Coregonus albula)* is natively distributed in Northern Europe (Kottelat and Freyhof 2007). In the northeast, the species' range overlaps with that of the Siberian cisco (*Coregonus sardinella*). Vendace from England and Scotland (*Coregonus vandensius*) is often regarded as conspecific to *C. albula* (Wheeler 1978; Reshentikov 2002), although Kottelat and Freyhof (2007) listed it as distinct together with additional European species in a so-called *C. albula* group. *Coregonus albula* is widely distributed in Sweden, including parts of the brackish Baltic Sea with low salinity. According to Lundberg (1899), it occurred naturally in *c*. 320 Swedish lakes, where after it has been introduced in *c*. 60 additional lakes (Filipsson 1994).

Ciscoes usually spawn during autumn from mid-October to mid-December, but spring-spawning (March to June) populations are known from four lakes in southern Sweden (Svärdson 1979) and two lakes in Northern Germany (Schulz and Freyhof 2003). The four Swedish spring-spawning populations from Lakes Ören, Åsunden, Stora Hålsjön, and Fegen were described as *C. trybomi* by Svärdson (1979), whereas in northeastern Germany, the spring-spawning forms from Lake Breiter Luzin and Lake Stechling have been described as *C. lucinensis* (Thienemann 1933) and *C. fontanae* (Schulz and Freyhof 2003), respectively. In addition, there are reports of spring- or winter (mid-December to mid-February)-spawning populations from Norway (Huitfeldt-Kaas 1927), Finland (Vuorinen et al. 1981), and Russia (Airaksinen 1968). Morphological differences described between spring or winter-spawners and their sympatric autumn-spawner include lower counts in spring/winter-spawners for certain meristic characters, and in two known cases, that is, Lake Fegen and Finnish Lake Änättijärvi, a larger eye (Airaksinen 1968; Svärdson 1979; Schulz and Freyhof 2003).

In the Swedish and German lakes with spring-spawning ciscoes, there is also a sympatric autumn-spawning population. In Finland, the situation is somewhat different with winter-spawning being the most common exception to the dominating autumn-spawning form, whereas spring-spawning has only been reported from two lakes (Vuorinen et al. 1981). Sympatric populations are known from three Finnish lakes, that is, one lake with spring-spawners and two with winter-spawners (Airaksinen 1968; Vuorinen et al. 1981).

According to Svärdson (1979, 1988), the occurrence of sympatric ciscoes in some Swedish lakes is a result of multiple invasions of already distinct species. However, this hypothesis has gained little or no support from allozyme studies that rather suggested independent local evolution of spring-spawners within each lake (Vourinen unpubl. data, cited in Svärdson 1988; Öst et al. 1990). Vuorinen et al. (1981) came to a similar conclusion regarding the Finnish sympatric populations and furthermore suggested that high water temperatures may promote local development of spawning periods shifted toward the winter or spring.

Schulz et al. (2006) used microsatellites and mtDNA sequences to study genetic relationships among the German spring-spawning *C. lucinencis* and *C. fontanea* and their sympatric autumn-spawning *C. albula* populations. They found lower genetic differentiation among the sympatric population pairs than between populations from different lakes, pointing toward independent origin of the two spring-spawning species. In line with this result, shared mtDNA haplotypes were observed in the sympatric species within each of the two German lakes, whereas they found no haplotype common to *C. fontanae* and *C. lucinensis*. In contrast, Mehner et al. (2010) found lower genetic differentiation between allopatric than sympatric populations in a follow-up study of the German *C. albula* complex using on a large AFLP marker panel. Schulz et al. (2006) also included mtDNA sequences from North American *C. sardinella* and detected evidence of past introgression into the two sympatric L. Breiter Luzin populations.

A recent review on the speciation of *Coregonus* (Hudson et al. 2005) suggested no less than six modes of speciation for sympatric forms. Compared to other *Coregonus* species complexes (e.g., whitefishes with up to five sympatric morphs/species), the *C*. *albula* group is less divergent. Resolving in further detail the diversity in this group could thus facilitate our understanding of the more complex diversification patterns observed in other coregonids. Inclusion of Swedish material is particularly needed to increase the amount of available genetic information, allowing different hypotheses to be tested regarding the present-day diversity in *C. albula*. So far, only two allozyme studies limited in scope have been carried out (Svärdson 1988; Öst et al. 1990). Three of the four spring-spawning populations in Sweden are regarded as extinct, and one spring-spawner (L. Fegen) has shown indications of decline. Notably, *C. trybomi* was previously listed as *Critically Endangered* in the Swedish red list, but due to uncertainties regarding its systematic status, it has recently been moved to the category *Data Deficient* (Gärdenfors 2010).

In this study, we analyzed mtDNA and microsatellite variation in spring- and autumn-spawning ciscoes from various parts of Sweden. Access to archived scales and frozen tissue allowed extraction of DNA from both extant and extinct populations. We investigated whether genetic variation in Swedish sympatric populations supports Svärdson's (1979) hypothesis involving secondary contact of already distinct forms, or more recent local diversification. The overall aim was to gain deeper understanding of postglacial immigration of the Northern European *C. albula* group. Some of the results should also be of importance for conservation and taxonomy.

## Materials and Methods

### Samples

**Figure 1:**
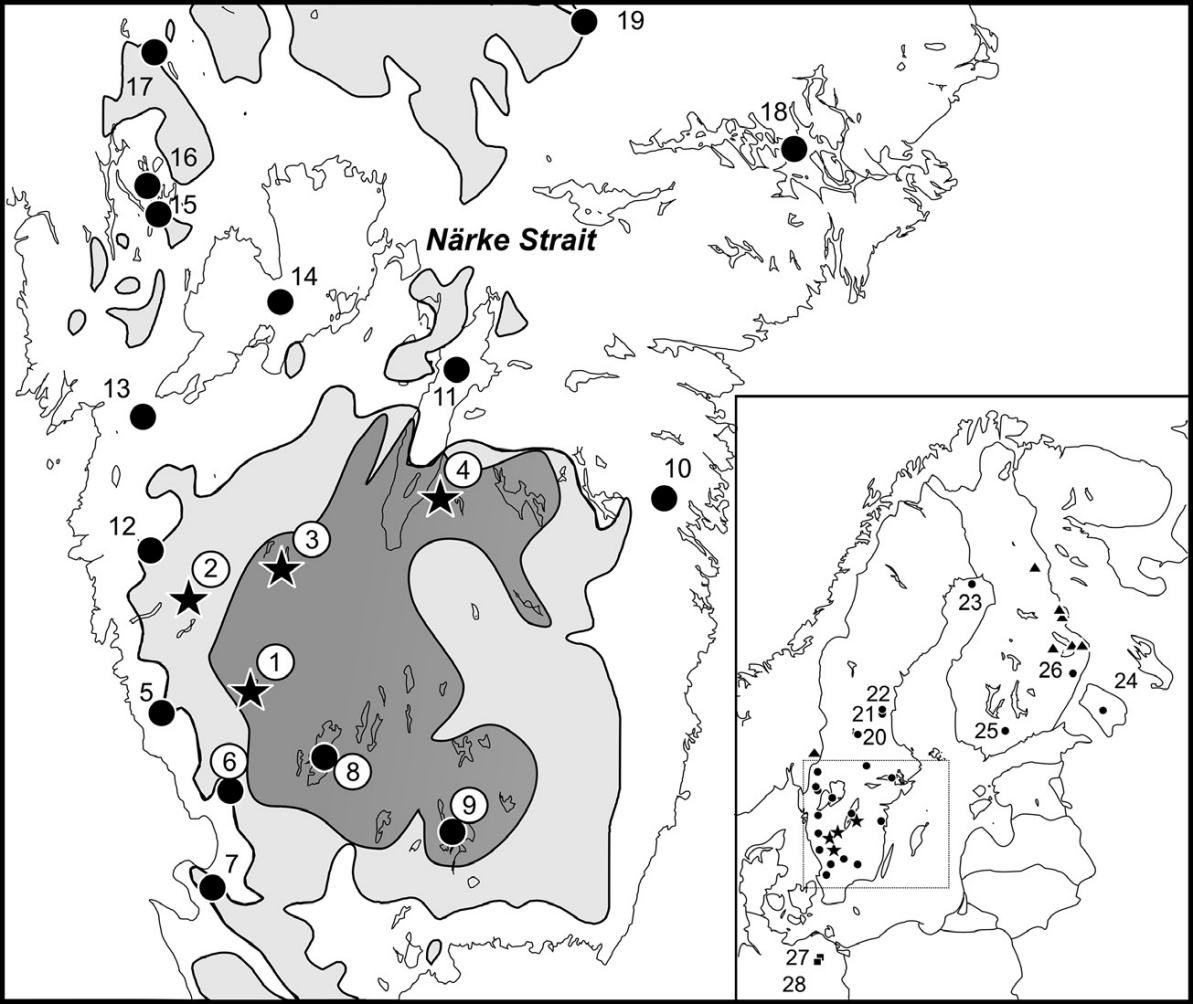
Sampling localities and localities mentioned in the text. Light gray indicates regions in Sweden above the highest shoreline of the Baltic basin, whereas dark gray indicates a region above highest shoreline temporarily covered by ice-dammed lakes. Stars and squares: Swedish and German lakes with sympatric spring- and autumn-spawners; dots: autumn-spawners only; triangles: one Norwegian and several Finnish lakes with winter- or spring-spawners. Numbers 1–24 refer to localities listed in Table 1; 25. Onkamo, 26. Kuohijärvi, 27. Breiter Luzin, 28. Stechlin. Encircled lakes (numbers) in Sweden belong to mtDNA Clade IA and microsatellite Assemblage I, possibly with exception for lake 6 (no microsatellite data).

Details on samples and populations analyzed in this study are given in Table 1 and Figure 1 (see also Supporting information). All ciscoes are of Swedish origin except for four specimens (two from the large-sized cisco *C. ladogae*, Lake Ladoga, Russia, and two *C. sardinella* from Northern Russia). Two specimens of *C. maraena* (Maraena whitefish) and one specimen of *Prosopium cylindraceum* (round whitefish) were included as outgroups. Apparently low mtDNA variation in the four Swedish target lakes with sympatric populations (Fegen, Ören, St. Hålsjön, and Åsunden) together with time-consuming protocols for old scales prompted a sampling strategy focused on small sample sizes from a larger number of populations (Table 1). Numbers of individuals analyzed with microsatellites were more extensive; those samples were originally collected for the purpose of studies on local fishery management and conservation (S. Palm, unpublished data) Most specimens with mtDNA sequences were also genotyped for microsatellites.

**Table 1:** Analyzed material of *Coregonus* spp. and *Prosopium cylindraceum*. Locality numbers correspond to Figure 1

		mtDNA				msat			
Locality	Locality	*N*	Voucher	Date	Tissue	*N*	Voucher	Date	Tissue
*Coregonus albula*									
Fegen (autumn-spawners)	1	2	NRM 54000	Aug 2003	Muscle in EtOH	70	SLU	Nov 2007, Nov 2008	Finclip in EtOH
Fegen (spring-spawners)	1	2	NRM 54007	Aug 2003	Muscle in EtOH	79	SLU	Apr and May 2008	Finclip in EtOH
Stora Hålsjön (autumn-spawners)	2	2	SLU	Nov 2005	Dry scales	50	SLU	May and Nov 2005	Finclip in EtOH
Stora Hålsjön (spring-spawners)	2	1,1^1^	SLU	Apr 1939	Dry scales				
Stora Hålsjön (autumn-spawners)	2	4^1^	SLU	Nov 1952	Dry scales				
Åsunden (autumn-spawners)	3	2	SLU	Nov 1968	Dry scales	29	SLU	May, Jul, Oct and Nov 2012	Finclip in EtOH
Åsunden (spring-spawners)	3	4^1^	SLU	May 1956	Dry scales				
Ören (autumn-spawners)	4	4	NRM 54994	May 2006	Muscle in EtOH	10	NRM 54994	May 2006	Finclip in EtOH
Ören (spring-spawners)	4	2	SU	May 1976	Frozen muscle				
Ören (spring-spawners)	4	6^1^	SLU	Apr 1957	Dry scales				
Ören (autumn-spawners)	4	2	SU	May 1976	Frozen muscle				
Skärsjön	5	4	SLU	Jul 2010	Dry scales				
Gyltigesjön	6	2	SLU	Aug 2008	Dry scales				
Rössjön	7	3	SLU	Dec 2007	Finclip in EtOH	25	SLU, NRM 65098 65100-104	Dec 2007, Sep 2009	Finclip in EtOH
Bolmen	8	4	SLU	Nov 2007	Finclip in EtOH	53	SLU, NRM65093	Nov 2007, Nov 2008	Finclip in EtOH
Åsnen	9	5	SLU	Sep 2007	Finclip in EtOH	40	SLU	2005, Sep and Nov 2007	Finclip in EtOH
Allgjuttern	10	3	SLU	Aug 2010	Dry scales				
Vättern	11	1,1^1^	NRM 57464, NRM 57465	Aug 2004	Muscle in EtOH				
Vättern	11	5	SLU	Aug 2009	Finclip in EtOH	32	SLU	Aug 2009	Finclip in EtOH
Stora Härsjön	12	2	SLU	Jul 2007	Dry scales				
Öresjö	13	2	SLU	Jul 2005	Dry scales				
Vänern (eastern part)	14	4	NRM 59932, 60090, 60021, 60018	Aug 2008	Muscle in EtOH				
Vänern (western part)	14	2	NRM 59954, 60017	Aug 2008	Muscle in EtOH	48	SLU	Aug 2009	Finclip in EtOH
Västra Solsjön	15	2	SLU	Jul 2004	Dry scales				
Västra Silen	16	2	NRM 57469	Nov 2006	Muscle in EtOH				
Ulvsjön	17	2	SLU	Jul 2003	Dry scales				
Mälaren (eastern part)	18	2	NRM 60038	Sep 2008	Muscle in EtOH				
Mälaren (eastern part)	18	2	SLU	Nov 2007	Finclip in EtOH	55	SLU, NRM 65096	Nov 2007	Finclip in EtOH
Mälaren (western part)	18	2	SLU	Nov 2007	Finclip in EtOH	55	SLU, NRM 65097	Nov 2007	Finclip in EtOH
Dagarn	19	3	SLU	Aug 2010	Dry scales				
Siljan	20	3	SLU	Sep 2009	Finclip in EtOH	31	SLU	Sep 2009	Finclip in EtOH
Södra Dellen	21	2	SLU	Nov 2007	Finclip in EtOH	39	SLU, NRM 65095	Nov 2007	Finclip in EtOH
Norra Dellen	22	2	SLU	Nov 2007	Finclip in EtOH	50	SLU, NRM 65094	Nov 2007	Finclip in EtOH
Kalix (Baltic Sea, Bothnian Bay)	23	6	SLU	Oct 2010	Finclip in EtOH	32	SLU	Oct 2010	Finclip in EtOH
Ladoga (*Coregonus ladogae*)	24	2	SLU	Autumn 2006	Finclip in EtOH				
Onkamo	25	3	From GenBank						
Kohijärvi	26	1	From GenBank						
Breiter Luzin (autumn-spawners)	27	3	From GenBank						
Breiter Luzin (*Coregonus lucinensis*)	27	4	From GenBank						
Stechlin (autumn-spawners)	28	2	From GenBank						
Stechlin (*Coregonus fontanane*)	28	5	From GenBank						
*Coregonus sardinella*									
Russia, Yamal Peninsula, river mouth		2	NRM 61262	Aug 1993	Frozen muscle				
Great Slave lake		1	From GenBank						
Avak River		1	From GenBank						
Shingle point		3	From GenBank						
*Coregonus maraena*									
Bolmen	8	2	SLU	Nov 2007	Finclip in EtOH	18	SLU	Nov 2007	Finclip in EtOH
*Prosopium cylindraceum*									
Russia, Anadyr, Tainorer River		1	NRM 57539	Aug 2005	Muscle in EtOH				
Total (Baltic ciscoes)		99				698			
Total (all species)		111				716			

*N*, number of analyzed individuals; NRM, Swedish Museum of Natural history; SLU, Swedish University of Agricultural Sciences (scale collection); SU, Stockholm University.

1 no DNA.

### Mitochondrial DNA analyses

DNA was extracted from muscle, fin tissue, and scales, and two mitochondrial DNA (mtDNA) fragments were amplified and sequenced, a part of the ND3 region and the D-loop of the control region (see supporting information for details). Our sequences were combined with already published ones from GenBank (Table S3, Supporting information). MrBayes v.3.2.1 (Ronquist et al. 2012) was used to infer phylogenetic relationships for the concatenated ND3 and D-loop regions. Two models of sequence evolution were selected by MrModeltest2 (Nylander 2004) as the best-fitting models of sequence evolution for each partition (HKY+G for the D-loop region and GTR+G for the ND3 region).

An unrooted network was constructed for the concatenated dataset using the software TCS (Clement et al. 2000). Phylogenetic nodes of interest were dated with BEAST v.1.7.5 (Drummond et al. 2012) assuming a strict molecular clock, a constant population size model, and with lower and upper limits of the range of substitution rate estimated by Crête-Lafrenière et al. (2012; 0.2%, 0.38% per million years [MY]). Tracer v.1.5 (Rambaut and Drummond 2007) was used to assess convergence for both phylogenetic analyses and to obtain dating estimates (Appendix S1, Supporting information).

### Microsatellite analyses

Total DNA was extracted following Walsh et al. (1991). A total of nine microsatellites found to be polymorphic in Baltic ciscoes and whitefish were genotyped: *BWF1, BWF2* (Patton et al. 1997), *Cisco90*, *Cisco126*, *Cisco157* (Turgeon et al., 1999), *Cocl23* (Bernatchez 1996), *Sfo8, Sfo23* (Angers et al. 1995), and *Str73* (Estoup et al. 1993). Details on PCR reactions are provided in Appendix S1 (Supporting information).

The genotypic data was checked with Microchecker 2.2.3 (Van Oosterhout et al. 2004) using a permutation procedure developed for identifying potential problems with stuttering, large allele dropouts, and null alleles. Fstat 2.9.3.2 (Goudet 1995) was used to compute unbiased estimates of heterozygosity, allelic richness, and *F*- and *R*-statistics, and to evaluate deviations from Hardy–Weinberg proportions and genotypic equilibrium. Fstat was also employed for *post hoc* comparisons of genetic variation within two groups of samples (Assemblages I & II; see below). A neighbor-joining tree (Saitou and Nei 1987) based on pairwise Cavalli-Sforza and Edwards (1967) chord distances was constructed with Phylip (Felsenstein 2005).

To evaluate the hypothesis that ciscoes in Sweden have a common postglacial origin, we assessed the relative importance of mutation and genetic drift behind the observed genetic structure using an allele-size-based randomization test (Hardy et al. 2003) implemented in SpaGeDi (Hardy and Vekemans 2002). Specifically, we tested whether *R*_ST_ > *F*_ST_, where *F*_ST_ (Weir and Cockerham 1984) measures genetic differentiation based on allele identity, whereas *R*_ST_ is based on allele size under a stepwise mutation model (Slatkin 1995; Michalakis and Excoffier 1996). If stepwise-like mutations have contributed significantly to the genetic divergence, *R*_ST_ is expected to be larger than *F*_ST_.

Divergence times between samples analyzed with microsatellites were assessed with model-based approximate Bayesian computation (ABC) as implemented in DIYABC 2.0 (Cornuet et al. 2008, 2010; submitted). Details on ABC analyses are provided in Appendix S2 (Supporting information). In brief, we estimated divergence time between two genetically distinct population assemblages (supported by mtDNA results). As a check, we also estimated the divergence time between the samples from Kalix (Baltic Sea) and Lake Vänern, two major water bodies which according to independent geological data became isolated c. 9000 yBP (Björk 1995).

## Results

### Mitochondrial genetic variation

**Figure 2:**
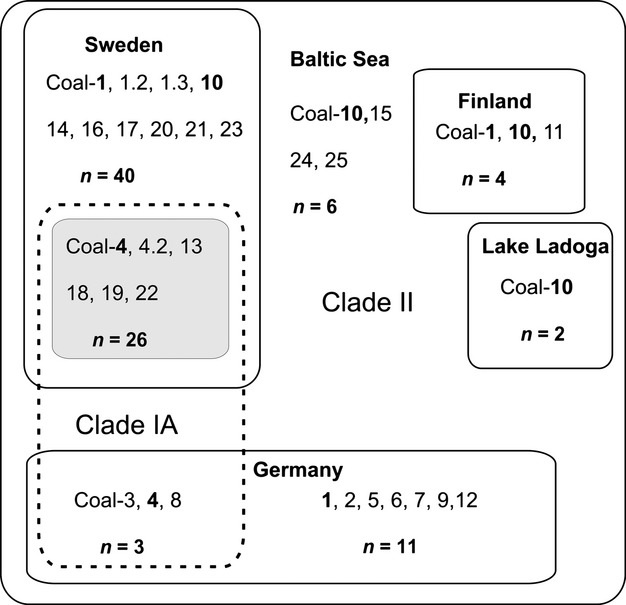
Schematic distribution of mtDNA haplotypes (ND3 + Dloop) hitherto found in different Northern European regions. Haplotype numbers in bold occur within more than one region. The grayed box represents southern Swedish lakes 1–4, 6, 8, and 9, associated with high altitude (cf. Fig. 1). Haplotypes in Clade IA are encircled with a dashed line to be distinct from those in Clade II. Sample size (n) refers to total number of sequenced specimens from each region. See Table S3 (Supporting information) for details.

Almost all muscle and fin tissue specimens yielded mtDNA sequences (98%). For dry scale specimens, the amplification success rate was lower (65%). The found haplotype distribution is summarized in Figure 2 and Table S3 (Supporting information). Compared to Schulz et al. (2006), slightly longer portions of both fragments were generated in this study (adding 23 and 14 bp for ND3 and D-loop, respectively). Novel haplotypes, only differing from previously published ones (e.g., Coal-1) in these extended regions, were named as Coal-1.2 and Coal-1.3.

We found previously undescribed haplotypes for both ND3 and D-loop, resulting in a total of 13 (16 with extended regions included) novel combined haplotypes for the Swedish material of *C. albula* (Tables S3 and S4, Supporting information). The Siberian *C. sardinella* samples also yielded two novel combined haplotypes.

**Figure 3:**
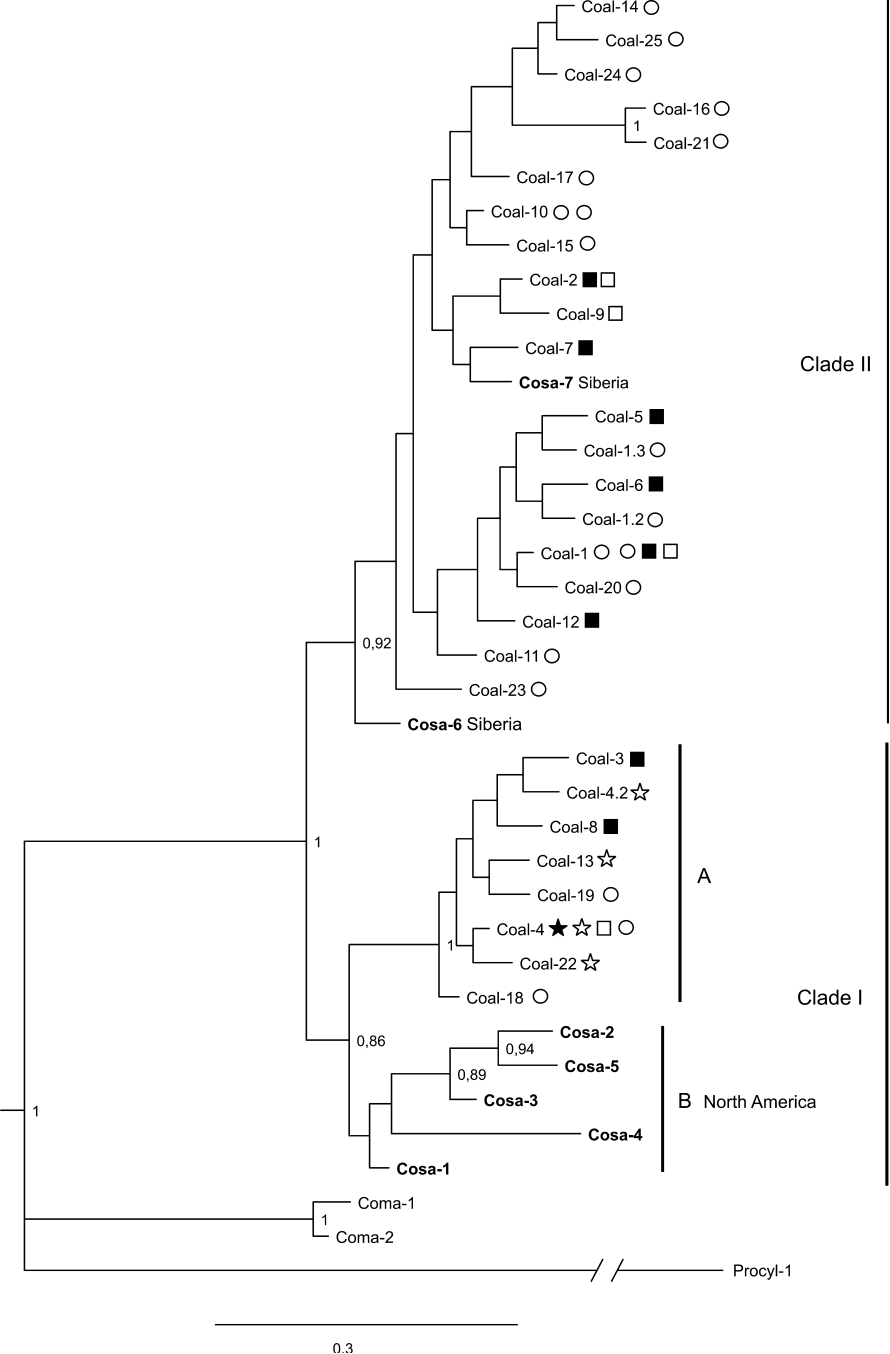
Bayesian phylogeny estimated from the concatenated ND3 and D-loop region of the mtDNA. Bayesian posterior probabilities above 0.85 are shown. Stars indicate presence of the haplotype in Swedish sympatric spring (filled)- and autumn-spawners (unfilled). Squares indicate presence of the haplotype in German sympatric spring (filled)- and autumn-spawners (unfilled). Circles indicate allopatric populations in Sweden (unfilled), Finland (gray), and Ladoga (gray).

Phylogenetic analysis of the mtDNA revealed two highly supported clades, from here on referred to as Clade I (further divided into subclades IA and IB) and Clade II (Fig. 3). The topology obtained from the phylogenetic analysis of the D-loop region alone (not shown) was identical to that in Figure 3 with regard to nodes leading to the major clades/subclades, whereas the topology recovered from the ND-3 region differed from that in Figure 3. However, most nodes in the separate ND3 and D-loop phylogenies were not statistically supported (Bayesian posterior probabilities below 0.5; Fig. S1, Supporting information) in contrast to the posterior probabilities of major nodes in Figure 3 (above 0.85), which indicates that each region alone is not as informative as the concatenated dataset for phylogenetic reconstruction.

Clade I has a wide geographical distribution in Sweden, Germany, and Northern America (Fig. 3). Subclade IA includes haplotypes from the four Swedish lakes with sympatric populations and other closely situated lakes. Haplotypes within this subclade also exist in spring- and autumn-spawners from German L. Breitzer Luzin (but not L. Stechlin), whereas subclade IB includes haplotypes found only in North American *C. sardinella*.

**Figure 4:**
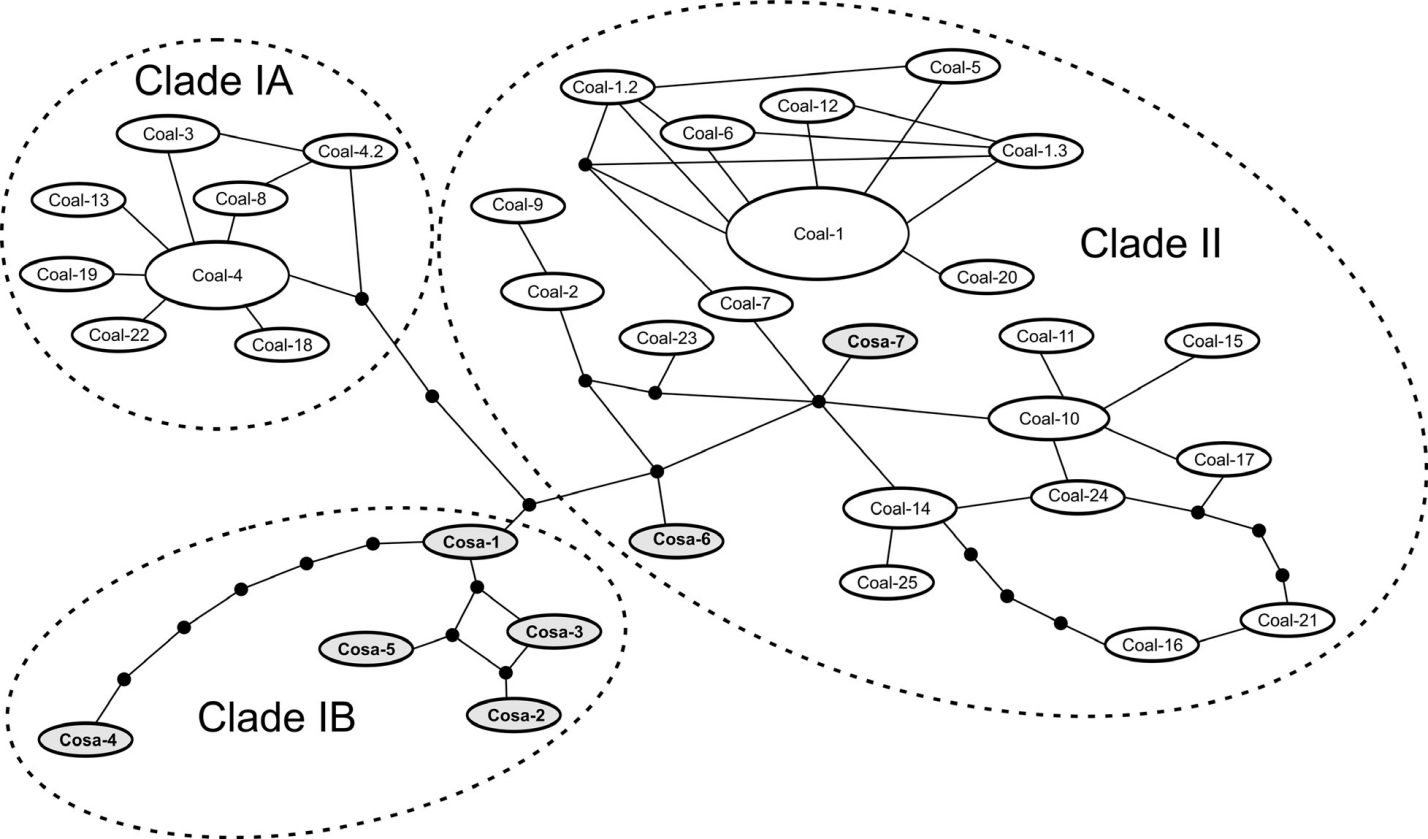
Statistic parsimony network of the concatenated ND3 and D-loop sequences with clades indicated as recovered by the phylogenetic analyses (Fig. 3).

Clade II (Fig. 3) consists of haplotypes present in all sympatric German cisco forms, and haplotypes within this clade are also distributed in Russia (L. Ladoga), Finland, and parts of Sweden (Fig. 2; Table S3). Notably, the two novel haplotypes found in *C. sardinella* from Siberia (Cosa–6 and Cosa–7) were placed within Clade II, in contrast to the North American *C. sardinella* haplotypes that are all in subclade IB (Figs. 4).

The Bayesian phylogeny estimated by BEAST (Fig. S2, Supporting information) was identical to the one obtained by MrBayes (Fig. 3) with respect to the two major clades supported by relatively high Bayesian posterior probabilities. The only difference was lack of support for subclade IB (Fig. S2, Supporting information). The age of the gene tree node that splits the two main mtDNA clades was estimated to be 2.4 MY and 4.5 MY, using a substitution rate of 0.38% and 0.2% per MY, respectively (95% highest posterior densities: 1.4–3.4 MY and 2.8–6.4 MY).

### Nuclear genetic variation

A total of 199 alleles were found at the nine microsatellites, ranging from 4 (*Str73)* to 51 (*BWF2)*, of which 27 (14 %) were private to a single sample. Bubble diagrams illustrating sample allele frequencies at each locus are shown in Fig. S3 (Supporting information). Average expected heterozygosity varied between 0.44 and 0.70, with corresponding estimates of allelic richness ranging from 3.2 to 6.9 (Table S6, Supporting information). Microchecker did not detect evidence of stuttering or large allele dropouts, whereas presence of a putative null allele(s) was indicated at a single locus in sample Kalix (*Sfo8*), E L. Mälaren (*Sfo23*), L. Siljan (*BWF1*), and L. Åsunden (*BWF1*).

Statistically significant deviations from Hardy–Weinberg proportions, quantified as average *F*_IS_ across loci, were found in three samples with an average heterozygote excess in L. Rössjön, whereas significant heterozygote deficiencies were detected in W L. Mälaren and L. Åsunden (Table S5, Supporting information). When testing for nonrandom association between genotypes at pairs of loci (genotypic disequilibrium), 22 of 537 tests (4%) within samples were significant (*P* < 0.05), close to the proportion expected by chance alone.

Pairwise sample estimates of *F*_ST_ and *R*_ST_ across all loci are given in Table S6 (Supporting information). Statistically significant genetic differentiation (*P* < 0.001) existed between the samples of sympatric spring- and autumn-spawners from L. Fegen, but without signs of a mutational component (*F*_ST_ = 0.063; *R*_ST_ = 0.049). In contrast, no significant allele frequency differences were found between the two samples from L. Mälaren (E vs. W) and L. Dellen (N vs. S; twin lakes connected by a short channel), respectively.

**Figure 5:**
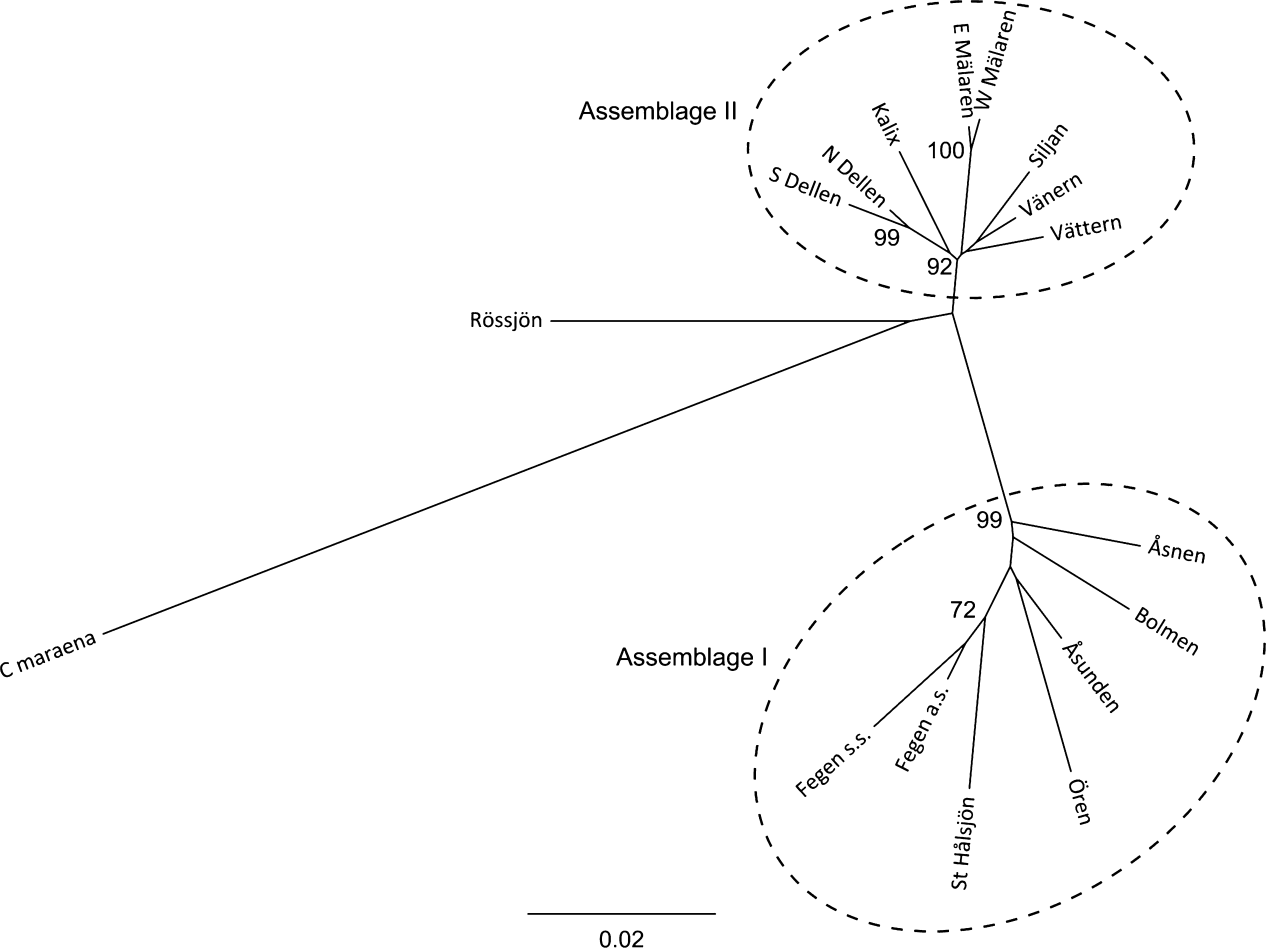
Unrooted neighbor-joining tree based on nine nuclear microsatellites and pairwise chord distances among 16 Baltic cisco populations and one sample of whitefish (*C. maraena*). Numbers are percentage node support values following 1000 bootstraps (only values >70% included).

Two distinct population groups with strong bootstrap supports, from here on referred to as Assemblage I and Assemblage II, were revealed by the neighbor-joining tree based on chord distance (Fig. 5). All sequenced individuals from Assemblage I exclusively carried haplotypes belonging to mtDNA Clade I and *vice versa* (Table S3, Supporting information; Fig. 3). The three sequenced individuals from L. Rössjön carried the same haplotype (Coal-14) belonging to Clade II. Hence, this sample was included in Assemblage II in the analyses below, despite lack of strong support in the neighbor-joining tree (Fig. 5).

When comparing the two population groups defined by congruent microsatellite variation, Assemblage II was found to have overall higher levels of genetic variability (Table S5, Supporting information); the differences were statistically significant with respect to both expected heterozygosity (*P* = 0.004) and allelic richness (*P* = 0.042; two-sided permutation tests with Fstat). Before testing, the genetically homogenous sample pairs from L. Mälaren and L. Dellen were lumped to avoid pseudoreplication.

Estimates of overall *F*_ST_ and *R*_ST_ across all samples per locus and in total are shown in Table 2. Random permutation of different allele sizes among allelic states revealed a significant mutational component to the overall differentiation found at three microsatellites (*BWF1*, *Cisco90*, *Sfo23*) and nearly across all nine loci (*P* = 0.06). Of all microsatellite alleles observed, 18% and 20% were private to samples in Assemblage I and Assemblage II, respectively (cf. Fig. S3, Supporting information).

**Table 2:** Overall nuclear genetic differentiation estimated with *F*_ST_ and *R*_ST_ between 14 Swedish Baltic cisco populations^1^

Locus	*F*_ST_	*R*_ST_	Permuted *R*_ST_ (95% CI)	*P*
*BWF1*	0.13	0.27	0.13 (0.04–0.28)	0.03
*BWF2*	0.15	0.15	0.14 (0.03–0.31)	0.38
*Cisco90*	0.18	0.37	0.17 (0.04–0.33)	0.01
*Cisco126*	0.19	0.20	0.16 (0.05–0.30)	0.31
*Cisco157*	0.12	0.07	0.11 (0.04–0.22)	0.68
*Cocl23*	0.21	0.22	0.20 (0.07–0.33)	0.41
*Sfo23*	0.03	0.12	0.03 (0.00–0.07)	0.00
*Sfo8*	0.13	0.16	0.12 (0.06–0.16)	0.07
*Str73*	0.10	0.08	0.09 (0.05–0.10)	0.83
All loci	0.14	0.18	0.12 (0.06–0.20)	0.06

An allele-size-based randomization test was used for evaluating whether or not *R*_ST_ (based on allele size) was significantly larger than *F*_ST_ (based on allele identity).

1 Genetically homogenous samples from E + W Mälaren and N + S Dellen were lumped.

Mean, median, and modal ABC estimates of divergence time between the ancestors of the two population assemblages based on microsatellite data (Appendix 2, Supporting information) ranged between *c*. 10,000–25,000 generations, corresponding to 40,000–100,000 yBP assuming a generation interval of 4 years (S. Palm, unpublished data). However, the estimate was associated with a considerable degree of uncertainty (95% c.i.: 3740–80,000 generations, or 15,000–320,000 yBP). The estimated divergence time between the genetically distinct populations (*P* < 0.001) from Kalix and L. Vänern (both in Assemblage II; *F*_ST_ = 0.019; *R*_ST_ = 0.003) ranged from *c*. 1700 to 4200 generations, or 6800–16,800 yBP (with a 95 % c.i.: 370–15,300 generations, or 1480–61,200 yBP).

## Discussion

Mitochondrial and nuclear DNA results were strikingly consistent regarding Swedish Baltic ciscoes, supporting two genetically distinct groups of local populations that appear fixed for mtDNA haplotypes from two different major clades. As shown by Schulz et al. (2006), Baltic cisco is paraphyletic in mtDNA with respect to *C. sardinella*, interpreted by them as past introgression into the *C. albula* complex. However, the inclusion of two additional samples of Siberian *C. sardinella* in this study together with a better supported phylogenetic tree (Fig. 3) revealed a more complex pattern, with the geographically remote North American *C*. *sardinella* haplotypes belonging to a distinct subclade distantly related to the two novel *C*. *sardinella* haplotypes from Siberia.

All Swedish spring-spawners were found to have haplotype Coal 4 from Clade IA, with their sympatric autumn-spawners carrying identical or almost identical haplotypes (only 1–2 mutations apart; Figs. 4). Moreover, according to genetic distances based on nuclear microsatellites, all four Swedish lakes with past or present sympatric populations belong to the same population assemblage (Fig. 5). Significant genetic differentiation was found between the only sympatric population pair analyzed for microsatellites (L. Fegen), but the differentiation was smaller than that among populations from different lakes regardless of spawning time (Table S6, Supporting information). Taken together, this indicates that all past and present sympatric population pairs in Sweden share a common origin associated with presence of *C*. *albula* Clade IA. Our results furthermore suggest that spring-spawning has evolved independently within each lake, similar to what has previously been proposed for sympatric populations in Finland (Vuorinen et al. 1981) and Germany (Schulz et al. 2006).

### Postglacial immigration

All Swedish populations in Assemblage I (microsatellites) with Clade IA haplotypes (mtDNA) are geographically restricted to a limited area in south-central Sweden (Fig. 1). This region is situated above the highest historic shoreline of the Baltic basin, but during early stages of the deglaciation, vast areas of the region were temporarily covered by ice-dammed lakes referred to as the South Swedish ice lake complex (Lundquist and Nilsson 1959; Donner 1995). Based on genetic data and the complex paleohydrological history of the Baltic basin at the end of the last glaciation, involving dramatic changes in salinity and sea/lake levels, we suggest a hypothesis that could explain the present-day distribution of mtDNA haplotypes and microsatellite alleles in Sweden.

A first “immigration wave,” from here on named Group I, may have arrived at the Baltic basin along the ice margin in Northern Europe, where it possibly reached the German lakes already some 12,000 yBP (cf. Schulz et al. 2006). Later, as the ice continued to retreat north, Group I spread in the Baltic Ice Lake and found its way up to the ice lake complex in southern Sweden. Svärdson (1988) has suggested a “sluicing-up mechanism” for how *Coregonus* may have spread to this region by means of ice readvances and temporarily ice-dammed lakes, which was originally proposed by Högbom (1917) to explain the occurrence of relict crustaceans in high-altitude lakes.

At about 10,600 yBP, the level of the Baltic Ice Lake dropped drastically, some 27–28 m, while draining to the west when the ice had retreaded north of Mt Billingen (Björk 1995). This drastic outflux of freshwater created a wide passage north of present Lake Vättern (the Närke Strait; Fig. 1) that allowed influx of salt sea water into the Baltic basin. The following brackish period (Yoldia Sea) lasted for about 1000 years, until the Baltic basin once again became a large freshwater lake (Ancylus Lake), which initially included the L. Vänern basin until the Närke Strait was again closed c. 9000 yBP (Björk 1995). Since about 8000 yBP, the Baltic basin has been brackish.

With a few exceptions (see below), the present-day distribution of Swedish populations, with Clade II mtDNA and microsatellite alleles belonging to Assemblage II, is restricted to water bodies at lower elevations associated with the Ancylus Lake (the Lake Vänern basin included). Thus, we suggest that the rapid lake level drop and marine influx in the Baltic basin c. 10,600 yBP may have suppressed the occurrence of the first arriving Group I in the Baltic basin, whereas a second immigration wave from another ice age refugium (Group II) arrived some 1000 years later in association with the formation of the Ancylus Lake. Depending on whether ciscoes remained in the Baltic basin or not, the later arriving Group II either recolonized all accessible areas or replaced (genetically swamped) Group I.

According to geological records, the three westernmost Swedish lakes studied herein, all with Clade II haplotypes (No. 5, 7 and 12; Fig. 1), were never in direct contact with the Ancylus Lake. Early arrival of Group II, already in the transition phase between Yoldia and Ancylus, could still explain these occurrences. During that period, a bay reached south in the present-day Kattegat Sea area where freshwater discharged from the Baltic Basin and from the surrounding mainland (Björk 1995). Hence, the salinity may have been low enough to allow colonization by ciscoes.

The more diverse haplotype distribution and complex haplotypic network within Clade II compared to Clade IA (Fig. 4) may suggest that the later colonizing Group II was further subdivided and/or had a larger effective population size before entering the Baltic Sea area. A corresponding pattern is also seen for the microsatellites, with a significantly higher level of genetic diversity within Assemblage II populations (Table S5, Supporting information). An alternative interpretation to the clear difference in genetic diversity levels could be a more recent genetic bottleneck associated with the colonization of the South Swedish ice lake complex. However, based on estimated ages of the splits between Assemblage I and Assemblage II and between Clade I and Clade II (see below), we find this explanation less likely.

The scenario proposed above, with two subsequent immigration events of genetically distinct ciscoes, is congruent with the present distribution of Clade II haplotypes in lower-altitude lakes – not only in Sweden but also according to the limited samples so far analyzed from Finland and Russian Lake Ladoga. Currently, the genetic origin of ciscoes in Finnish lakes with spring/winter-spawning populations remains unknown, and unfortunately, all Finnish populations with deviating spawning period seem to have become extinct (K.-J.M. Himberg, pers. com.). Thus, future genetic studies should focus on remaining autumn-spawners in those Finnish lakes to assess whether a genetic pattern similar to the one in Sweden could exists. Previous allozyme data (Vuorinen et al. 1981) did not support a genetic dichotomy between lakes with spring/winter-spawning populations and lakes with autumn-spawners only. We note, however, that the Finnish lakes with spring/winter-spawning populations are all situated at high altitude compared to lakes with autumn-spawners only (see Table 1 in Vuorinen et al. 1981). This was also noted by Airaksinen (1968) who pointed out that all those lakes are situated above the highest coast line of the Baltic Ice Lake. Furthermore, several temporal ice-dammed lakes existed in that region of Finland (Donner 1995), which could have functioned as pathways to high-altitude lakes.

Evidence of subsequent immigration events in Northern Europe also exist for several other freshwater organisms. Segerstråle (1957) described and discussed the distribution of relict crustaceans and one fish (fourhorn sculpin) in relation to postglacial colonization of the Baltic basin from the northeast by means of sluicing-up mechanisms (*sensu* Högbom 1917), and recognized two groups of species characterized by differences in maximum altitude below the highest coastline. In addition, recent genetic studies of several freshwater fishes in Northern Europe have suggested multiple colonization events (e.g., perch, grayling, Atlantic salmon; Nesbø et al. 1999; Koskinen et al. 2002; Säisä et al. 2005). However, none of these studies is easily comparable to our results, because samples from high-altitude localities in southern Sweden have not been included or because the species in question is not natively distributed in that region.

In the southern Baltic area, Schulz et al. (2006) found a mix of both Clade I and Clade II haplotypes in L. Breiter Luzin, whereas in L. Stechlin only Clade II haplotypes were observed. The difference between Sweden and Germany with respect to spatial distributions of the two main mtDNA clades offers at least two different explanations: either an effect of past stocking activities in these German lakes (Mehner et al. 2009, 2010), or natural colonization. The latter explanation was suggested by Mehner et al. (2010), who proposed that their results, combined with those in Schulz et al. (2006), could be explained by introgression from a second lineage that arrived later. Brzuzan et al. (2002) also studied mtDNA variation in *C. albula* from five lakes in neighboring Poland, using RFLP analysis of the ND3, ND4, and D-loop regions. Although RFLP results are not directly comparable to sequence data, their haplotypes formed two distinct groups in an UPGMA phenogram which may correspond to Clade IA and Clade II. Furthermore, although both groups of RFLP haplotypes occurred mixed in all but one of the studied populations, Brzuzan et al. (2002) could see a tendency for regional differences in relative haplotype frequencies and discussed this pattern in relation to possible postglacial immigration from different refugia.

### Divergence times

Some of the most variable microsatellites display a high proportion of private alleles within the two assemblages, and signs of a significant mutational component to population divergence were also obtained (i.e., *R*_ST_ > *F*_ST_), supporting a longer time of independent diversification. In line with these findings, the ABC estimate of divergence time among the two Swedish population assemblages suggested a split between their ancestors that coincides roughly with the onset of the last glaciation (some 100,000 yBP), although we note that this time estimate is associated with a wide credibility interval. The second ABC analysis, including L. Vänern and Kalix (Baltic Sea), suggested a much later population split close to the estimated geological date for the closure of the Närke strait separating the two basins (i.e., c. 9000 yBP). Although this second estimate is also uncertain, its correlation with independent geological data may indicate that the above ABC estimate of divergence time (between the two major population assemblages) could be of a correct order of magnitude.

In contrast, the Bayesian estimates for split times of the major mtDNA clades were significantly larger, that is, several millions of years. However, besides that sequence divergence is often older than population divergence (e.g., Edwards and Beerli 2000), our time estimates based on mtDNA data should be interpreted with caution. First, they rely solely on the implemented rate of substitution, which in turn is dependent on several critical assumptions (see Crête-Lafrenière et al. 2012 for details). Second, Bayesian coalescent methods may lead to biased estimates of divergence time in cases of population structure or a complex demographic history (Navascués and Emerson 2009). Third, substitution rates estimated across short-term evolutionary time spans have been found to be significantly higher than established long-term (phylogenetic) mutation rates; a pattern suggested to reflect purifying selection (Ho et al. 2005).

Because the implemented mtDNA substitution rate in Salmonidae was calibrated using fossils (Crête-Lafrenière et al. 2012), it is possible that it has been underestimated compared to the true substitution rate and, thus, that our current split time estimates for the two major clades in the mtDNA gene tree are biased upward (cf. Audzijonyte and Väinölä 2006). This could potentially explain the large discrepancy between the time estimates obtained from the two dating analyses employed in this study. Hence, we suggest that the true split time of the two mtDNA clades could be younger than the time inferred herein. However, further genetic data including more accurate estimates of mutation rates will be needed to resolve the divergence times between the mitochondrial clades.

### Taxonomy and conservation

Focusing on the *C. albula* group as a whole, also including C. *sardinella*, and accounting for estimated ages of population assemblages and mtDNA clades, it seems that either (i) extensive introgression occurred during the Pleistocene, involving populations of *C*. *albula* and *C. sardinella* as we recognize them today, or that (ii) the current mixture of haplotypes is a result of incomplete lineage sorting among ancestors to the two present species. The answer could also be a combination of these two processes, as their distinction is not always clear cut. Regardless, the present results reveal that *C. albula* and *C. sardinella,* in some way, have had extensive contact back in time.

Paleohydrological conditions during recurrent Pleistocene glaciations most certainly played an important role for present-day distribution of Coregonid fishes, for example, the disjunct distribution of *Stenodus* in the Arctic and Caspian Sea (Reshentikov 2002), the close morphological and genetic affinity between *C. autumnalis* in Siberia and *C*. *pollan* in Ireland (Ferguson et al. 1978), and the *C. albula* complex as shown herein. However, suggesting possible pathways for past genetic exchange between North America and Europe across Asia is not easy based on available paleohydrological maps and accounting for maximal extensions of ice sheets and ice-dammed lakes during the last glaciation (cf. Grosswald 1980). It also would be premature based on the limited material studied genetically so far. Furthermore, it seems fully possible that the split between Clade I and II could have predated the last glaciation.

The taxonomic position of *C*. *sardinella* as a distinct species has seldom been doubted, as it is characterized by several phenotypic characters (e.g., smaller head, larger size, and semianadromous life history). However, we note that Yakhnenko and Mamontov (2002) discussed a small lake-resident spring-spawning form of *C. sardinella* from the Lena River basin that resembled *C. albula* in morphology. Furthermore, Borovikova and Makhrov (2009) found a haplotype in the mitochondrial ND1-gene in *C. albula* from Lake Vodlozero (Baltic basin) that was more similar to Siberian *C. sardinella* haplotypes than it was to those typically found in *C. albula*. Hence, a comprehensive molecular and morphological study, including East European and Siberian material combined with expanded sampling from North (and Western) Europe appears warranted for a proper genetic and taxonomic distinction of *C. sardinella* vs. *C. albula*, including also doubtful taxa like *C. ladogae* (L. Ladoga) and *C. kiletz* (L. Onega), and for providing a better understanding of the group's complex evolutionary history.

With respect to spring-spawning ciscoes in Sweden, the present results do not support the previous recognition of *C. trybomi* as a distinct species (Svärdson 1979). The only extant spring-spawning population in L. Fegen rather seems to constitute one out of several populations in southern Sweden with a common phylogeographic origin. Nevertheless, the spring-spawning L. Fegen population with its rare life history and distinctive morphology (Svärdson 1979) should be protected in its local environment. Furthermore, we do not exclude that the ability to switch spawning time could be a trait unique to the population assemblage to which the four known cases belong. Hence, all remaining Swedish Group I populations may arguably be of particular value for conservation regardless of their spawning time.

## Data Accessibility

All sequences have been deposited in GenBank under accession numbers: KJ830843-KJ830845, KJ830848-KJ830849, KJ830855, KJ830858-KJ830870, KJ83076-KJ830883, KJ830886-KJ830887, KJ830893, KJ830896-KJ830898, and KJ830914-KJ830918.

The microsatellite data have been deposited at Dryad: doi: 10.5061/dryad.67cn1.

## Supporting Information

**Appendix S1.** Material and methods. **Table S1.** Details of the primers used in this study for amplification of the mitochondrial ND-3 region and D-loop region. **Table S2.** GenBank accession numbers of already published sequences. **Table S3.** Frequency distribution of haplotypes in *C. albula* and *C*. *sardinella*. **Table S4.** Variable positions for the ND3-region and the D-loop segments. **Table S5.** Genetic variation in samples analyzed with nine nuclear microsatellites. **Table S6.** Pairwise sample estimates with *F*_ST_ and *R*_ST_ based on nine microsatellites. **Figure S1.** Bayesian phylogeny estimated from the ND3 region of the mtDNA. **Figure S2.** Dated Bayesian phylogeny estimated from the concatenated ND3 and D-loop region of the mtDNA. **Figure S3.** Bubble diagrams illustrating relative allele frequencies at nine microsatellite loci in Swedish ciscoes. **Appendix S2.** ABC analyses. **Table S7.** Priors for demographic model parameters. **Table S8.** Priors for mutational model parameters (divided at two “locus groups”). **Table S9.** Estimated posterior parameter distributions based on 10,000 selected datasets. **Figure S4.** Simple demographic model used for two ABC analyses. **Figure S5.** Principal component analyses based on summary statistics for real data, simulated prior and posterior parameter distributions.
