# Supplementary material for: Genetic signs of multiple colonization events in Baltic ciscoes with radiation into sympatric spring- and autumn-spawners confined to early postglacial arrival
Source: Ecol Evol. 2014 Oct 27;4(22):4346–60. doi: 10.1002/ece3.1299 (PMC4267872; doi:10.1002/ece3.1299)
Supplement: Supplementary file 1 [file ece30004-4346-SD1.docx]

Delling *et al*. – Supporting information

**Appendix S1**

**Material and methods**

All Swedish cisco populations in the present study are assumed to be indigenous and unaffected by stocking, except for L. Gyltigesjön that originates from an unknown introduction before year 1970 (Filipsson 1994). However, this sample was retained as part of the studied material with this clarification.

Sympatric spring and autumn spawners were collected during spawning in April/May and November respectively, and only mature individuals were included. However, two exceptions to this rule exist (Table 1); to separate spring from autumn spawners in the L. Fegen sample from August 2003, a diagnostic morphological difference among the two sympatric populations (confirmed with genetic data; Delling unpublished, Palm et al. in prep.) was used, whereas the L. Ören sample from May 2006 (autumn spawners) consisted of non-mature adults collected after the presumed extinction of the local spring spawning population.

*Mitochondrial DNA extraction and sequencing*DNA was extracted from muscle tissue, fin tissue and scales (n=88, Table 1) using the MoleStrips DNA tissue kit on a GeneMole® automated DNA extraction instrument following the protocol provided by the manufacturer. Muscle and fin tissues stored in ethanol were dried at room temperature before DNA extraction. All samples were incubated overnight at 55ºC with lysis buffer and proteinase K. For dry scales, 80mM (final concentration) of dithiothreitol (DTT) were added in the lysis solution.

Two mitochondrial DNA (mtDNA) fragments were amplified for all specimens that successfully yielded DNA; a 266bp fragment of the ND-3 region and a 400bp fragment of the D-loop of the control region. The first fragment was amplified using primers ND-3-FOR and ND-3-REW from Schulz *et al*. (2006). The second fragment was amplified with primers developed specifically for this study (primer pair D-loop in Table S1).

Due to the degraded nature of DNA in the dry scales specimens, we re-designed primers to target amplicons shorter than 200bp for both mtDNA fragments. Hence, for dry scale specimens the ND-3 region was amplified in two overlapping fragments (primer pairs ND-3-a, ND-3-b in Table S1) and the D-loop region in three overlapping fragments (D-loop-a, D-loop-b, D-loop-c in Table S1). Moreover, we developed primers that target the ND-3 region of the *Prosopium cylindraceum* specimen (primer pair ND-3 prosopium in Table S1)to use this species as an outgroup in phylogenetic analyses.

Polymerase chain reactions (PCR) for mtDNA were performed using the illustra Hot Start Mix RTG from GE Healthcare Life Sciences, which contains a mix of freeze-dried PCR reagents including PuReTaq DNA polymerase and a hot start activator protein. Purified water, forward and reverse primers in a final concentration of 0.2μM each, and 2μl of DNA extract were added to the mix in a final volume of 25μl. PCR conditions were 15 min at 95ºC, followed by 28 or 40 cycles (fresh samples and dry scales, respectively) of 1 min at 95ºC, 45 sec at T_anneal_ (see Table S1 for annealing temperatures for different primer pairs), 1 min at 72ºC, and a final extension step of 10 min at 72ºC. PCR products were purified using Exonuclease I and FastAP Thermosensitive Alkaline Phosphatase and sequenced in both directions on an ABI3130xl (Applied Biosystems). Sequences were assembled and aligned in Geneious 5.0.1 (Drummond *et al*. 2012). All mtDNA sequencing was performed at the Laboratory of Molecular Systematics, Swedish Museum of Natural History.

*Testing for saturation of mtDNA*

Each partition of the concatenated dataset was assessed for saturation using the test of substitution saturation in DAMBE v.5.3.108 (Xia et al. 2003; Xia and Lemey 2009). The ND3 region was analyzed for the first and second codon positions and the third codon position separately. The results showed that each partition have experienced little substitution saturation, which does not affect their phylogenetic signal.

*Microsatellite genotyping*

All microsatellite loci were co-amplified in a multiplex PCR reaction using Ready-To-Go PCR beads (GE Healthcare) and approximately 100 ng of template DNA. Primers were end-labeled with fluorescent dyes as to enable co-migration of all loci in the same capillary during electrophoresis. The PCR amplification was initiated with a denaturation step at 94°C for 5 min, followed by 30 cycles of 30 s at 94°C, 30 s at 53°C and 1 min at 72°C. The process was terminated with a 10 min elongation step at 72ºC. Electrophoresis was made on an ABI Prism 310 Genetic Analyzer (Applied Biosystems, Foster City, California, USA). Allele sizes were determined with Genotyper 3.7 (Applied Biosystems). All microsatellite analyses were performed at the Institute of Freshwater Research, Drottningholm.

**Table S1.** Details of the primers used in this study for amplification of the mitochondrial ND-3 region and D-loop region.

| **Primer pair** | **Forward sequence 5’-3’** | **Reverse sequence 5’-3’** | **T_anneal_ (ºC)** | **Product size (bp)** |
| --- | --- | --- | --- | --- |
| ND-3* | CATCACCATCGCACTATCCA | CCTCCTTGGGTTCACTCGTA | 48 | 266 |
| D-loop | CCCACCCTTAACTCCCAAAG | TCACGAGATGCTACCTAATACGA | 53 | 400 |
| ND-3-a | CATCACCATCGCACTATCCA | GCGATCTCTAGGTCAAATAA | 50 | 146 |
| ND-3-b | GACTACCTTTTTCCCTGCGC | CCTCCTTGGGTTCACTCGTA | 52 | 147 |
| D-loop-a | CCCACCCTTAACTCCCAAAG | AGGATGTACTACCCACGTGC | 52 | 132 |
| D-loop-b | TGCATATTATGTACTGACCC | GTGTTAGCTGGAGGTTTGTTG | 50 | 151 |
| D-loop-c | CACCAACGGAACCGTTCTAA | TCACGAGATGCTACCTAATACGA | 52 | 147 |
| ND-3  Prosopium | ATGAACTTAATCACGACAG | CTGCCTATTCAGCTCATTC | 48 | 318 |

** primer sequences from Schulz et al. (2006)*

*T_anneal_ (ºC): annealing temperature*

*Product size excluding primers*

**Table S2.** GenBank accession numbers of already published sequences used in this study.

| **Accession number** | **Species** | **Locus** |
| --- | --- | --- |
| AY277989 | *Coregonus albula* | D-loop |
| AY277991 | *Coregonus albula* | D-loop |
| AY277994 | *Coregonus sardinella* | D-loop |
| AY277995 | *Coregonus sardinella* | D-loop |
| AY277996 | *Coregonus sardinella* | D-loop |
| AY277997 | *Coregonus sardinella* | D-loop |
| AY277998 | *Coregonus sardinella* | D-loop |
| AY278000 | *Coregonus albula* | ND-3 |
| AY278002 | *Coregonus albula* | ND-3 |
| AY278007 | *Coregonus albula* | ND-3 |
| AY278010 | *Coregonus albula* | ND-3 |
| AY278011 | *Coregonus albula* | ND-3 |
| AY278013 | *Coregonus albula* | ND-3 |
| AY278016 | *Coregonus albula* | ND-3 |
| AY278017 | *Coregonus sardinella* | ND-3 |
| AY278018 | *Coregonus sardinella* | ND-3 |
| AY278019 | *Coregonus sardinella* | ND-3 |
| AY278020 | *Coregonus sardinella* | ND-3 |
| AY278021 | *Coregonus sardinella* | ND-3 |

**Table S3** Frequency distribution of haplotypes in *C. albula* (Coal-1 to Coal-25) and *C*. *sardinella* (Cosa-1 to Cosa-7) sorted by clade. Frequencies given in bold for the concatenated sequences (ND3 + D-loop), within () for ND3 only.

|  |  | ***C. albula*** | | | | | | | | | | | | | | | | | | | | | | | | | | | | | | | ***C. sardinella*** | | | | | | | |
| --- | --- | --- | --- | --- | --- | --- | --- | --- | --- | --- | --- | --- | --- | --- | --- | --- | --- | --- | --- | --- | --- | --- | --- | --- | --- | --- | --- | --- | --- | --- | --- | --- | --- | --- | --- | --- | --- | --- | --- | --- |
|  |  | **IA** | | | | | | | | **II** | | | | | | | | | | | | | | | | | | | | | | | **IB** | | | | | **II** | | |
|  |  | 3 | 4 | 4.2 | 8 | 13 | 18 | 19 | 22 | | 1 | 1.2 | 1.3 | 2 | 5 | 6 | 7 | 9 | 10 | 11 | 12 | 14 | 15 | 16 | 17 | 20 | 21 | 23 | 24 | 25 |  | 1 | | 2 | 3 | 4 | 5 | | 6 | 7 |
| **Locality** | **No** |  |  |  |  |  |  |  |  | |  |  |  |  |  |  |  |  |  |  |  |  |  |  |  |  |  |  |  |  |  |  | |  |  |  |  | |  |  |
| ***Coregonus albula*** |  |  |  |  |  |  |  |  |  | |  |  |  |  |  |  |  |  |  |  |  |  |  |  |  |  |  |  |  |  |  |  | |  |  |  |  | |  |  |
| Fegen (autumn spawners) | 1 | - | **1** | **-** | **-** | **1** | **-** | **-** | **-** | | **-** | **-** | **-** | **-** | **-** | **-** | **-** | **-** | **-** | **-** | **-** | **-** | **-** | **-** | **-** | **-** | **-** | **-** | **-** | **-** |  | - | | - | - | - | - | | - | - |
| Fegen (spring spawners) |  | - | **2** | **-** | **-** | **-** | **-** | **-** | **-** | | **-** | **-** | **-** | **-** | **-** | **-** | **-** | **-** | **-** | **-** | **-** | **-** | **-** | **-** | **-** | **-** | **-** | **-** | **-** | **-** |  | - | | - | - | - | - | | - | - |
| Stora Hålsjön (autumn spawners) | 2 | - | **2** | **-** | **-** | **-** | **-** | **-** | **-** | | **-** | **-** | **-** | **-** | **-** | **-** | **-** | **-** | **-** | **-** | **-** | **-** | **-** | **-** | **-** | **-** | **-** | **-** | **-** | **-** |  | - | | - | - | - | - | | - | - |
| Stora Hålsjön (spring spawners) |  | - | **1** | **-** | **-** | **-** | **-** | **-** | **-** | | **-** | **-** | **-** | **-** | **-** | **-** | **-** | **-** | **-** | **-** | **-** | **-** | **-** | **-** | **-** | **-** | **-** | **-** | **-** | **-** |  | - | | - | - | - | - | | - | - |
| Åsunden (autumn spawners) | 3 | - | **1** | **-** | **-** | **-** | **-** | **-** | **1** | | **-** | **-** | **-** | **-** | **-** | **-** | **-** | **-** | **-** | **-** | **-** | **-** | **-** | **-** | **-** | **-** | **-** | **-** | **-** | **-** |  | **-** | | **-** | **-** | **-** | **-** | | **-** | **-** |
| Ören (autumn spawners) | 4 | - | **3** | **1** | **-** | **-** | **-** | **-** | **-** | | **-** | **-** | **-** | **-** | **-** | **-** | **-** | **-** | **-** | **-** | **-** | **-** | **-** | **-** | **-** | **-** | **-** | **-** | **-** | **-** |  | **-** | | **-** | **-** | **-** | **-** | | **-** | **-** |
| Ören (spring spawners) |  | - | **2** | **-** | **-** | **-** | **-** | **-** | **-** | | **-** | **-** | **-** | **-** | **-** | **-** | **-** | **-** | **-** | **-** | **-** | **-** | **-** | **-** | **-** | **-** | **-** | **-** | **-** | **-** |  | **-** | | **-** | **-** | **-** | **-** | | **-** | **-** |
| Skärsjön | 5 | - | **-** | **-** | **-** | **-** | **-** | **-** | **-** | | **-** | **-** | **-** | **-** | **-** | **-** | **-** | **-** | **-** | **-** | **-** | **1** | **-** | **-** | **-** | **1** | **2** | **-** | **-** | **-** |  | **-** | | **-** | **-** | **-** | **-** | | **-** | **-** |
| Gyltigesjön | 6 | - | **2** | **-** | **-** | **-** | **-** | **-** | **-** | | **-** | **-** | **-** | **-** | **-** | **-** | **-** | **-** | **-** | **-** | **-** |  | **-** | **-** | **-** | **-** | **-** | **-** | **-** | **-** |  | **-** | | **-** | **-** | **-** | **-** | | **-** | **-** |
| Rössjön | 7 | - | **-** | **-** | **-** | **-** | **-** | **-** | **-** | | **-** | **-** | **-** | **-** | **-** | **-** | **-** | **-** | **-** | **-** | **-** | **3** | **-** | **-** | **-** | **-** | **-** | **-** | **-** | **-** |  | **-** | | **-** | **-** | **-** | **-** | | **-** | **-** |
| Bolmen | 8 | - | **2** | **-** | **-** | **-** | **1** | **1** | **-** | | **-** | **-** | **-** | **-** | **-** | **-** | **-** | **-** | **-** | **-** | **-** | **-** | **-** | **-** | **-** | **-** | **-** | **-** | **-** | **-** |  | **-** | | **-** | **-** | **-** | **-** | | **-** | **-** |
| Åsnen | 9 | - | **5** | **-** | **-** | **-** | **-** | **-** | **-** | | **-** | **-** | **-** | **-** | **-** | **-** | **-** | **-** | **-** | **-** | **-** | **-** | **-** | **-** | **-** | **-** | **-** | **-** | **-** | **-** |  | **-** | | **-** | **-** | **-** | **-** | | **-** | **-** |
| Allgjuttern | 10 | - | **-** | **-** | **-** | **-** | **-** | **-** | **-** | | **3** |  | **-** | **-** | **-** | **-** | **-** | **-** | **-** | **-** | **-** | **-** | **-** | **-** | **-** | **-** | **-** | **-** | **-** | **-** |  | **-** | | **-** | **-** | **-** | **-** | | **-** | **-** |
| Vättern | 11 | - | **-** | **-** | **-** | **-** | **-** | **-** | **-** | | **5** | **1** | **-** | **-** | **-** | **-** | **-** | **-** | **-** | **-** | **-** | **-** | **-** | **-** | **-** | **-** | **-** | **-** | **-** | **-** |  | **-** | | **-** | **-** | **-** | **-** | | **-** | **-** |
| Stora Härsjön | 12 | - | **-** | **-** | **-** | **-** | **-** | **-** | **-** | | **1** | **-** | **-** | **-** | **-** | **-** | **-** | **-** | **-** | **-** | **-** | **-** |  | **1** | **-** | **-** | **-** | **-** | **-** | **-** |  | **-** | | **-** | **-** | **-** | **-** | | **-** | **-** |
| Öresjö | 13 | - | **-** | **-** | **-** | **-** | **-** | **-** | **-** | | **1** | **-** | **-** | **-** | **-** | **-** | **-** | **-** | **1** | **-** | **-** | **-** | **-** | **-** | **-** | **-** | **-** | **-** | **-** | **-** |  | **-** | | **-** | **-** | **-** | **-** | | **-** | **-** |
| Vänern (eastern part) | 14 | - | **-** | **-** | **-** | **-** | **-** | **-** | **-** | | **2** | **-** | **-** | **-** | **-** | **-** | **-** | **-** | **1** | **-** | **-** | **-** | **-** | **-** | **-** | **-** | **-** | **1** | **-** | **-** |  | **-** | | **-** | **-** | **-** | **-** | | **-** | **-** |
| Vänern (western part) |  | - | **-** | **-** | **-** | **-** | **-** | **-** | **-** | | **1** | **-** | **-** | **-** | **-** | **-** | **-** | **-** | **1** | **-** | **-** | **-** | **-** | **-** | **-** | **-** | **-** | **-** | **-** | **-** |  | **-** | | **-** | **-** | **-** | **-** | | **-** | **-** |
| Västra Solsjön | 15 | - | **-** | **-** | **-** | **-** | **-** | **-** | **-** | | **2** | **-** | **-** | **-** | **-** | **-** | **-** | **-** | **-** | **-** | **-** | **-** | **-** | **-** | **-** | **-** | **-** | **-** | **-** | **-** |  | **-** | | **-** | **-** | **-** | **-** | | **-** | **-** |
| Västra Silen | 16 | - | **-** | **-** | **-** | **-** | **-** | **-** | **-** | | **-** | **-** | **-** | **-** | **-** | **-** | **-** | **-** | **2** | **-** | **-** | **-** | **-** | **-** | **-** | **-** | **-** | **-** | **-** | **-** |  | **-** | | **-** | **-** | **-** | **-** | | **-** | **-** |
| Ulvsjön | 17 | - | **-** | **-** | **-** | **-** | **-** | **-** | **-** | | **2** | **-** | **-** | **-** | **-** | **-** | **-** | **-** | **-** | **-** | **-** | **-** | **-** | **-** | **-** | **-** | **-** | **-** | **-** | **-** |  | **-** | | **-** | **-** | **-** | **-** | | **-** | **-** |
| Mälaren (eastern part) | 18 | - | **-** | **-** | **-** | **-** | **-** | **-** | **-** | | **1** | **-** | **-** | **-** | **-** | **-** | **-** | **-** | **2** | **-** | **-** | **1** | **-** | **-** | **-** | **-** | **-** | **-** | **-** | **-** |  | **-** | | **-** | **-** | **-** | **-** | | **-** | **-** |
| Mälaren (western part) |  | - | **-** | **-** | **-** | **-** | **-** | **-** | **-** | | **2** | **-** | **-** | **-** | **-** | **-** | **-** | **-** | **-** | **-** | **-** | **-** | **-** | **-** | **-** | **-** | **-** | **-** | **-** | **-** |  | **-** | | **-** | **-** | **-** | **-** | | **-** | **-** |
| Dagarn | 19 | - | **-** | **-** | **-** | **-** | **-** | **-** | **-** | | **2** | **-** | **1** | **-** | **-** | **-** | **-** | **-** | **-** | **-** | **-** | **-** | **-** | **-** | **-** | **-** | **-** | **-** | **-** | **-** |  | **-** | | **-** | **-** | **-** | **-** | | **-** | **-** |
| Siljan | 20 | - | **-** | **-** | **-** | **-** | **-** | **-** | **-** | | **2** | **-** | **-** | **-** | **-** | **-** | **-** | **-** | **-** | **-** | **-** | **-** | **-** | **-** | **-** | **-** | **-** | **-** | **-** | **-** |  | **-** | | **-** | **-** | **-** | **-** | | **-** | **-** |
| Södra Dellen | 21 | - | **-** | **-** | **-** | **-** | **-** | **-** | **-** | | **-** | **-** | **-** | **-** | **-** | **-** | **-** | **-** | **-** | **-** | **-** | **-** | **-** | **1** | **1** | **-** | **-** | **-** | **-** | **-** |  | **-** | | **-** | **-** | **-** | **-** | | **-** | **-** |
| Norra Dellen | 22 | - | **-** | **-** | **-** | **-** | **-** | **-** | **-** | | **-** | **-** | **-** | **-** | **-** | **-** | **-** | **-** | **-** | **-** | **-** | **2** | **-** | **-** | **-** | **-** | **-** | **-** | **-** | **-** |  | **-** | | **-** | **-** | **-** | **-** | | **-** | **-** |
| Kalix (Baltic Sea, Bothnian Bay) | 23 | - | **-** | **-** | **-** | **-** | **-** | **-** | **-** | | **-** | **-** | **-** | **-** | **-** | **-** | **-** | **-** | **3** | **-** | **-** | **-** | **1** | **-** | **-** | **-** | **-** | **-** | **1** | **1** |  | **-** | | **-** | **-** | **-** | **-** | | **-** | **-** |
| Ladoga (*C. ladogae*) | 24 | - | **-** | **-** | **-** | **-** | **-** | **-** | **-** | | **-** | **-** | **-** | **-** | **-** | **-** | **-** | **-** | **2** | **-** | **-** | **-** | **-** | **-** | **-** | **-** | **-** | **-** | **-** | **-** |  | **-** | | **-** | **-** | **-** | **-** | | **-** | **-** |
| Onkamo | 25 | - | **-** | **-** | **-** | **-** | **-** | **-** | **-** | | **1** | **-** | **-** | **-** | **-** | **-** | **-** | **-** | **1**(6) | **1**(2) | **-** | **-** | **-** | **-** | **-** | **-** | **-** | **-** | **-** | **-** |  | **-** | | **-** | **-** | **-** | **-** | | **-** | **-** |
| Kuhojärvi | 26 | - | **-** | **-** | **-** | **-** | **-** | **-** | **-** | | **-** | **-** | **-** | **-** | **-** | **-** | **-** | **-** | **1**(4) | **-** | **-** | **-** | **-** | **-** | **-** | **-** | **-** | **-** | **-** | **-** |  | **-** | | **-** | **-** | **-** | **-** | | **-** | **-** |
| Breiter Luzin (autumn spawners) | 27 | - | **1** | **-** | **-** | **-** | **-** | **-** | **-** | | **1**(2) | **-** | **-** | **1**(16) | **-** | **-** | **-** | **1** | **-** | **-** | **-** | **-** | **-** | **-** | **-** | **-** | **-** | **-** | **-** | **-** |  | **-** | | **-** | **-** | **-** | **-** | | **-** | **-** |
| (spring spawners; *C. lucinensis*) |  | **1**(11) | **-** | **-** | **1** | **-** | **-** | **-** | **-** | | **-** | **-** | **-** | **1**(8) | **-** | **-** | **-** | **-** | **-** | **-** | **-** | **-** | **-** | **-** | **-** | **-** | **-** | **-** | **-** | **-** |  | **-** | | **-** | **-** | **-** | **-** | | **-** | **-** |
| Stechlin (autumn spawners) | 28 | **-** | **-** | **-** | **-** | **-** | **-** | **-** | **-** | | **1**(14) | **-** | **-** | **1**(5) | **-** | **-** | **-** | **-** | **-** | **-** | **-** | **-** | **-** | **-** | **-** | **-** | **-** | **-** | **-** | **-** |  | **-** | | **-** | **-** | **-** | **-** | | **-** | **-** |
| (spring spawners; *C. fontanae*) |  | **-** | **-** | **-** | **-** | **-** | **-** | **-** | **-** | | **1**(11) | **-** | **-** | **-** | **1**(2) | **1** | **1**(2) | **-** | **-** | **-** | **1** | **-** | **-** | **-** | **-** | **-** | **-** | **-** | **-** | **-** |  | **-** | | **-** | **-** | **-** | **-** | | **-** | **-** |
|  |  |  |  |  |  |  |  |  |  | |  |  |  |  |  |  |  |  |  |  |  |  |  |  |  |  |  |  |  |  |  |  | |  |  |  |  | |  |  |
|  |  |  |  |  |  |  |  |  |  | |  |  |  |  |  |  |  |  |  |  |  |  |  |  |  |  |  |  |  |  |  |  | |  |  |  |  | |  |  |
| **Table S3** Continued | | | | | | | | | | | | | | | | | | | | | | | | | | | | | | | | | | | | | | | | |
| ***Coregonus sardinella*** |  | **-** | **-** | **-** | **-** | **-** | **-** | **-** | **-** | | **-** | **-** | **-** | **-** | **-** | **-** | **-** | **-** | **-** | **-** | **-** | **-** | **-** | **-** | **-** | **-** | **-** | **-** | **-** | **-** |  | **-** | | **-** | **-** | **-** | **-** | | - | - |
| Russia, Yamal Peninsula |  | **-** | **-** | **-** | **-** | **-** | **-** | **-** | **-** | | **-** | **-** | **-** | **-** | **-** | **-** | **-** | **-** | **-** | **-** | **-** | **-** | **-** | **-** | **-** | **-** | **-** | **-** | **-** | **-** |  | **-** | | **-** | **-** | **-** | **-** | | **1** | **1** |
| Great Slave lake |  | **-** | **-** | **-** | **-** | **-** | **-** | **-** | **-** | | **-** | **-** | **-** | **-** | **-** | **-** | **-** | **-** | **-** | **-** | **-** | **-** | **-** | **-** | **-** | **-** | **-** | **-** | **-** | **-** |  | **-** | | **1** | **-** | **-** | **-** | | **-** | **-** |
| Avak River |  | **-** | **-** | **-** | **-** | **-** | **-** | **-** | **-** | | **-** | **-** | **-** | **-** | **-** | **-** | **-** | **-** | **-** | **-** | **-** | **-** | **-** | **-** | **-** | **-** | **-** | **-** | **-** | **-** |  | **1** | | **-** | **-** | **-** | **-** | | **-** | **-** |
| Shingle point |  | **-** | **-** | **-** | **-** | **-** | **-** | **-** | **-** | | **-** | **-** | **-** | **-** | **-** | **-** | **-** | **-** | **-** | **-** | **-** | **-** | **-** | **-** | **-** | **-** | **-** | **-** | **-** | **-** |  | **-** | | **-** | **1** | **1** | **1** | | **-** | **-** |

**Table S4** Variable positions for the ND3-region and the D-loop segments relative to the sequence Coal-1. Positions marked with an asterisk (*) and written in *italics* refers to parts of the sequences outside previously available haplotypes in Genbank. Position numbers are not synchronized to fit the already published ones.

|  | *** | **ND3 position** | | | |  |  |  |  |  |  |  |  |  |  |  |  |  | *** | *** |  | **D-loop position** | | | | |  |  |  |  |  |  |  |  |  |
| --- | --- | --- | --- | --- | --- | --- | --- | --- | --- | --- | --- | --- | --- | --- | --- | --- | --- | --- | --- | --- | --- | --- | --- | --- | --- | --- | --- | --- | --- | --- | --- | --- | --- | --- | --- |
| **Haplotype** | *0* | 0 | 0 | 0 | 0 | 1 | 1 | 1 | 1 | 1 | 1 | 1 | 1 | 1 | 2 | 2 | 2 |  | *0* | *0* | 0 | 0 | 0 | 1 | 1 | 1 | 1 | 1 | 2 | 2 | 2 | 2 | 2 | 2 | 2 |
|  | *1* | 3 | 5 | 5 | 9 | 4 | 4 | 5 | 5 | 6 | 6 | 7 | 7 | 8 | 0 | 1 | 2 |  | *1* | *2* | 0 | 2 | 2 | 0 | 0 | 1 | 2 | 7 | 0 | 2 | 4 | 4 | 5 | 8 | 9 |
|  | *5* | 4 | 0 | 2 | 4 | 5 | 8 | 5 | 7 | 5 | 8 | 3 | 8 | 7 | 9 | 1 | 7 |  | *2* | *0* | 4 | 4 | 7 | 0 | 5 | 5 | 5 | 4 | 8 | 2 | 1 | 3 | 9 | 9 | 7 |
| **Coal-1** | ***G*** | **A** | **T** | **A** | **T** | **T** | **A** | **G** | **C** | **C** | **C** | **A** | **A** | **T** | **T** | **G** | **A** |  | ***A*** | ***T*** | **A** | **G** | **C** | **T** | **A** | **G** | **C** | **G** | **T** | **G** | **C** | **A** | **G** | **A** | **A** |
| **Coal-1.2** | **-** | **-** | **-** | **-** | **-** | **-** | **-** | **-** | **-** | **-** | **-** | **-** | **-** | **-** | **-** | **-** | **-** |  | *-* | ***C*** | **-** | **-** | **-** | **-** | **-** | **-** | **-** | **-** | **-** | **-** | **-** | **-** | **-** | **-** | **-** |
| **Coal-1.3** | **-** | **-** | **-** | **-** | **-** | **-** | **-** | **-** | **-** | **-** | **-** | **-** | **-** | **-** | **-** | **-** | **-** |  | ***G*** | ***-*** | **-** | **-** | **-** | **-** | **-** | **-** | **-** | **-** | **-** | **-** | **-** | **-** | **-** | **-** | **-** |
| **Coal-2** | **-** | **-** | **C** | **-** | **-** | **-** | **-** | **-** |  | **G** | **-** | **-** | **-** | **-** | **-** | **-** | **-** |  | **-** | **-** | **-** | **-** | **-** | **-** | **-** | **-** | **-** | **-** | **-** | **-** | **-** | **-** | **-** | **-** | **-** |
| **Coal-3** | **-** | **-** | **C** | **-** | **C** | **-** | **-** | **-** | **-** | **-** | **-** | **-** | **-** | **-** | **-** | **-** | **-** |  | **-** | **-** | **-** | **A** | **-** | **C** | **-** | **-** | **-** | **-** | **C** | **-** | **A** | **G** | **-** | **-** | **G** |
| **Coal-4** | **-** | **-** | **C** | **-** | **C** | **-** | **-** | **-** | **-** | **-** | **-** | **-** | **-** | **-** | **-** | **-** | **-** |  | **-** | **-** | **-** | **A** | **-** | **C** | **-** | **-** | **-** | **-** | **C** | **-** | **A** | **G** | **-** | **-** | **-** |
| **Coal-4.2** | **-** | **-** | **C** | **-** | **C** | **-** | **-** | **-** | **-** | **-** | **-** | **-** | **-** | **-** | **-** | **-** | **-** |  | *-* | ***C*** | - | **A** | **-** | **C** | **-** | **-** | **-** | **-** | **C** | **-** | **A** | **G** | **-** | **-** | **-** |
| **Coal-5** |  | **-** | **-** | **-** | **-** | **C** | **-** | **-** | **-** | **-** | **-** | **-** | **-** | **-** | **-** | **-** | **-** |  |  | **-** | **-** | **-** | **-** | **-** | **-** | **-** | **-** | **-** | **-** | **-** | **-** | **-** | **-** | **-** | **-** |
| **Coal-6** |  | **-** | **-** | **-** | **-** | **-** | **-** | **-** | **-** | **-** | **-** | **-** | **-** | **-** | **-** | **-** | **-** |  |  | **-** | **-** | **-** | **-** | **-** | **-** | **-** | **-** | **-** | **-** | **-** | **-** | **-** | **-** | **-** | **-** |
| **Coal-7** |  | **-** | **C** | **-** | **-** | **-** | **-** | **-** | **-** | **-** | **-** | **-** | **-** | **-** | **-** | **-** | **-** |  |  | **-** | **-** | **-** | **-** | **-** | **-** | **A** | **-** | **-** | **-** | **-** | **-** | **-** | **-** | **-** | **-** |
| **Coal-8** |  | **-** | **C** | **G** | **C** | **-** | **-** | **-** | **-** | **-** | **-** | **-** | **-** | **-** | **-** | **-** | **-** |  |  | **-** | **-** | **A** | **-** | **C** | **-** | **-** | **-** | **-** | **C** | **-** | **A** | **G** | **-** | **-** | **-** |
| **Coal-9** |  | **-** | **C** | **-** | **-** | **-** | **-** | **-** |  | **G** | **-** | **-** | **-** | **C** | **-** | **-** | **-** |  |  | **-** | **-** | **-** | **-** | **-** | **-** | **-** | **-** | **-** | **-** | **-** | **-** | **-** | **-** | **-** | **-** |
| **Coal-10** | **-** | **G** | **C** | **-** | **-** | **-** | **-** | **-** | **-** | **-** | **-** | **-** | **-** | **-** | **-** | **-** | **-** |  | **-** | **-** | **-** | **-** | **-** | **-** | **-** | **-** | **-** | **-** | **-** | **-** | **-** | **-** | **-** | **-** | **-** |
| **Coal-11** |  | **G** | **C** | **-** | **-** | **-** | **-** | **-** | **-** | **-** | **-** | **G** | **-** | **-** | **-** | **-** | **-** |  |  | **-** | **-** | **-** | **-** | **-** | **-** | **-** | **-** | **-** | **-** | **-** | **-** | **-** | **-** | **-** | **-** |
| **Coal-12** |  | **-** | **-** | **-** | **-** | **-** | **-** | **-** | **-** | **-** | **-** | **G** | **-** | **-** | **-** | **-** | **-** |  |  | **-** | **-** | **-** | **-** | **-** | **-** | **-** | **-** | **-** | **-** | **-** | **-** | **-** | **-** | **-** | **-** |
| **Coal-13** | **-** | **-** | **C** | **-** | **C** | **-** | **-** | **-** | **-** | **-** | **-** | **-** | **-** | **-** | **-** | **-** | **-** |  | **-** | **-** | **-** | **A** | **-** | **C** | **-** | **-** | **-** | **-** | **C** | **C** | **A** | **G** | **-** | **-** | **-** |
| **Coal-14** | **-** | **-** | **C** | **-** | **-** | **-** | **-** | **-** | **-** | **-** | **-** | **-** | **-** | **-** | **-** | **-** | **-** |  | **-** | **-** | **-** | **-** | **-** | **-** | **-** | **-** | **-** | **-** | **C** | **-** | **-** | **-** | **-** | **-** | **-** |
| **Coal-15** | **-** | **G** | **C** | **-** | **-** | **-** | **-** | **-** | **-** | **-** | **A** | **-** | **-** | **-** | **-** | **-** | **-** |  | **-** | **-** | **-** | **-** | **-** | **-** | **-** | **-** | **-** | **-** |  | **-** | **-** | **-** | **-** | **-** | **-** |
| **Coal-16** | ***C*** | **-** | **C** | **-** | **-** | **-** | **G** | **A** | **-** | **-** | **-** | **-** | **-** | **-** | **-** | **-** | **-** |  | **-** | **-** | **-** | **-** | **-** | **-** | **-** | **-** | **-** | **-** | **C** | **-** | **-** | **-** | **A** | **-** | **-** |
| **Coal-17** | **-** | **G** | **C** | **-** | **-** | **-** | **-** | **-** | **-** | **-** | **-** | **-** | **-** | **-** | **-** | **-** | **-** |  | **-** | **-** | **-** | **-** | **-** | **-** | **-** | **-** | **-** | **-** |  | **-** | **-** | **-** | **A** | **-** | **-** |
| **Coal-18** | **-** | **G** | **C** | **-** | **C** | **-** | **-** | **-** | **-** | **-** | **-** | **-** | **-** | **-** | **-** | **-** | **-** |  | **-** | **-** | **-** | **A** | **-** | **C** | **-** | **-** | **-** | **-** | **C** | **-** | **A** | **G** | **-** | **-** | **-** |
| **Coal-19** | **-** | **-** | **C** | **-** | **C** | **-** | **-** | **-** | **-** | **-** | **-** | **-** | **-** | **-** | **-** | **-** | **-** |  | **-** | **-** | **-** | **A** | **-** | **A** | **-** | **-** | **-** | **-** | **C** | **-** | **A** | **G** | **-** | **-** | **-** |
| **Coal-20** | **-** | **-** | **-** | **-** | **-** | **-** | **-** | **-** | **-** | **-** | **-** | **-** | **-** | **-** | **-** | **-** | **-** |  | **-** | **-** | **-** | - | **-** | - | **-** | **-** | **-** | **A** | **-** | **-** | **-** | **-** | **-** | **-** | **-** |
| **Coal-21** | ***C*** | **G** | **C** | **-** | **-** | **-** | **G** | **A** | **-** | **-** | **-** | **-** | **-** | **-** | **-** | **-** | **-** |  | **-** | **-** | **-** | - | **-** | - | **-** | **-** | **-** | **-** | **C** | **-** | **-** | **-** | **A** | **-** | **-** |
| **Coal-22** | **-** | **-** | **C** | **-** | **C** | **-** | **-** | **-** | **-** | **-** | **-** | **-** | **G** | **-** | **-** | **-** | **-** |  | **-** | **-** | **-** | **A** | **-** | **C** | **-** | **-** | **-** | **-** | **C** | **-** | **A** | **G** |  | **-** | **-** |
| **Coal-23** | **-** | **-** | **C** | **-** | **-** | **-** | **-** | **-** | **-** | **-** | **-** | **-** | **-** | **-** | **C** | **-** | **-** |  | **-** | **-** | **-** | **C** | **-** | **-** | **-** | **-** | **-** | **-** | **-** | **-** | **-** | **-** | **-** | **-** | **-** |
| **Coal-24** | **-** | **G** | **C** | **-** | **-** | **-** | **-** | **-** | **-** | **-** | **-** | **-** | **-** | **-** | **-** | **-** | **-** |  | **-** | **-** | **-** | **-** | **-** | **-** | **-** | **-** | **-** | **-** | **C** | **-** | **-** | **-** | **-** | **-** | **-** |
| **Coal-25** | **-** | **-** | **C** | **-** | **-** | **-** | **-** | **-** | **-** | **-** | **-** | **-** | **-** | **-** | **-** | **-** | **-** |  | **-** | **-** | **-** | - | **T** | - | **-** | **-** | **-** | **-** | **-** | **-** | **-** | **-** | **-** | **-** | **-** |
| **Cosa-1** |  | **-** | **C** | **-** | **C** | **-** | **-** | **-** | **-** | **-** | **-** | **-** | **-** | **-** | **-** | **-** | **-** |  |  | **-** | **-** | **A** | **-** | **C** | **-** | **-** | **-** | **-** | **-** | **-** | **-** | **-** | **-** | **-** | **-** |
| **Cosa-2** |  | **-** | **C** | **-** | **C** | **-** | **G** | **-** | **-** | **-** | **-** | **G** | **-** | **-** | **-** | **-** | **-** |  |  | **-** | **-** | **A** | **-** | **C** | **C** |  | **A** | **-** | **-** | **-** | **-** | **-** | **-** | **-** | **-** |
| **Cosa-3** |  | **-** | **C** | **-** | **C** | **-** | **G** | **-** | **-** | **-** | **-** | **G** | **-** | **-** | **-** | **-** | **-** |  |  | **-** | **-** | **A** | **-** | **C** | **-** | **-** | **-** | **-** | **-** | **-** | **-** | **-** | **-** | **-** | **-** |
| **Cosa-4** |  | **-** | **C** | **-** | **C** | **-** | **-** | **-** | **-** | **-** | **-** | **-** | **-** | **-** | **-** | **A** | **-** |  |  | **-** | **-** | **A** | **-** | **C** | **-** | **-** | **-** | **-** | **-** | **-** | **-** | **-** | **-** | **C** | **-** |
| **Cosa-5** |  | **-** | **C** | **-** | **C** | **-** | **G** | **-** | **-** | **-** | **-** | **-** | **-** | **-** | **-** | **-** | **C** |  |  | **-** | **-** | **A** | **-** | **C** | **-** | **-** | **A** | **-** | **-** | **-** | **-** | **-** | **-** | **-** | **-** |
| **Cosa-6** | **-** | **-** | **C** | **-** | **C** | **-** | **-** | **-** | **-** | **-** | **-** | **-** | **-** | **-** | **-** | **-** | **-** |  | *-* | *-* | **G** | - | **-** | **-** | **-** | **-** | **-** | **-** | **-** | **-** | **-** | **-** | **-** | **-** | **-** |
| **Cosa-7** | **-** | **-** | **C** | **-** | **-** | **-** | **-** | **-** | **-** | **-** | **-** | **-** | **-** | **-** | **-** | **A** | **-** |  | **-** | **-** | **-** | **-** | **-** | **-** | **-** | **-** | **-** | **-** | **-** | **-** | **-** | **-** | **-** | **-** | **-** |

**Table S5** Genetic variation in samples analyzed with nine nuclear microsatellites: *n*= sample size (no. individuals); *H_E_* = average expected heterozygosity (gene diversity); *A_R_* = average allelic richness (20 genes per sample); *F*_IS_ = measure of Hardy-Weinberg conformance (average across all nine loci) with level of significance. Assemblage I and II refer to clusters of genetically similar populations (cf. Figure 5). a.s.=autumn spawners, s.s.=spring spawners.

| Sample | | *n* | *H_E_* | *A_R_* (20 genes) | *F*_IS_ |  |
| --- | --- | --- | --- | --- | --- | --- |
|  |  |  |  |  |  |  |
| *Population assemblage I* | | |  |  |  |  |
|  | Bolmen (a.s.) | 53 | 0.554 | 5.2 | -0.003 |  |
|  | Fegen (a.s.) | 70 | 0.484 | 4.8 | -0.011 |  |
|  | Fegen (s.s.) | 79 | 0.479 | 4.0 | 0.022 |  |
|  | St Hålsjön (a.s.) | 50 | 0.448 | 4.0 | -0.008 |  |
|  | Åsnen (a.s.) | 40 | 0.520 | 5.6 | -0.005 |  |
|  | Åsunden (a.s.) | 29 | 0.585 | 5.3 | 0.112 | ** |
|  | Ören (a.s.) | 10 | 0.518 | 4.6 | 0.036 |  |
|  |  |  |  |  |  |  |
| *Population assemblage II* | | |  |  |  |  |
|  | S Dellen (a.s.) | 39 | 0.645 | 6.3 | 0.006 |  |
|  | N Dellen (a.s.) | 50 | 0.667 | 6.7 | -0.023 |  |
|  | Kalix (a.s.) | 32 | 0.661 | 6.9 | 0.045 |  |
|  | E Mälaren (a.s.) | 55 | 0.655 | 5.7 | 0.033 |  |
|  | W Mälaren (a.s.) | 55 | 0.664 | 5.5 | 0.069 | ** |
|  | Rössjön (a.s.) | 25 | 0.437 | 3.3 | -0.112 | * |
|  | Siljan (a.s.) | 31 | 0.677 | 6.6 | -0.005 |  |
|  | Vänern (a.s.) | 48 | 0.700 | 6.5 | -0.019 |  |
|  | Vättern (a.s.) | 32 | 0.672 | 6.8 | 0.018 |  |
|  |  |  |  |  |  |  |
| *Average/total* | | | | | |  |
|  | Assemblage I | 331 | 0.512 | 4.8 | 0.011 |  |
|  | Assemblage II | 367 | 0.642 | 6.0 | 0.011 |  |
|  |  |  |  |  |  |  |
|  | All samples | 698 | 0.585 | 5.5 | 0.011 |  |
|  |  |  |  |  |  |  |

* *P*<0.05; ** *P*<0.01

**Table S6** Pairwise sample estimates with *F*_ST_ (above diagonal) and *R*_ST_ (below diagonal) based on nine microsatellites. Bold *F*_ST_ values represent comparisons not associated with a significant allele frequency difference (underlined bold values having *P*>0.05 even before Bonferroni correction (*k*=120) for multiple tests).

|  |  | Assemblage I | | | | | | |  | Assemblage II | | | | | | | | |
| --- | --- | --- | --- | --- | --- | --- | --- | --- | --- | --- | --- | --- | --- | --- | --- | --- | --- | --- |
|  |  | Bolmen | Fegen AS | Fegen SS | St Hålsjön | Åsnen | Åsunden | Ören |  | S Dellen | N Dellen | Kalix | E Mälaren | W Mälaren | Rössjön | Siljan | Vänern | Vättern |
|  |  |  |  |  |  |  |  |  |  |  |  |  |  |  |  |  |  |  |
| Assemblage I | Bolmen |  | 0.122 | 0.187 | 0.162 | 0.146 | 0.092 | 0.103 |  | 0.170 | 0.155 | 0.158 | 0.167 | 0.173 | 0.305 | 0.139 | 0.129 | 0.142 |
|  | Fegen AS | 0.091 |  | 0.063 | 0.143 | 0.147 | 0.065 | 0.097 |  | 0.177 | 0.158 | 0.165 | 0.197 | 0.203 | 0.328 | 0.202 | 0.159 | 0.171 |
|  | Fegen SS | 0.237 | 0.049 |  | 0.154 | 0.173 | 0.079 | 0.134 |  | 0.186 | 0.174 | 0.175 | 0.214 | 0.215 | 0.328 | 0.221 | 0.174 | 0.191 |
|  | St Hålsjön | 0.238 | 0.121 | 0.174 |  | 0.134 | 0.122 | 0.163 |  | 0.194 | 0.181 | 0.183 | 0.215 | 0.219 | 0.353 | 0.211 | 0.163 | 0.175 |
|  | Åsnen | 0.147 | 0.044 | 0.100 | 0.067 |  | 0.112 | 0.119 |  | 0.142 | 0.123 | 0.094 | 0.091 | 0.110 | 0.301 | 0.138 | 0.088 | 0.096 |
|  | Åsunden | 0.233 | 0.138 | 0.210 | 0.089 | 0.180 |  | 0.044 |  | 0.110 | 0.094 | 0.105 | 0.135 | 0.141 | 0.269 | 0.112 | 0.088 | 0.097 |
|  | Ören | 0.115 | -0.001 | 0.082 | 0.043 | 0.044 | 0.035 |  |  | 0.145 | 0.133 | 0.123 | 0.152 | 0.164 | 0.323 | 0.156 | 0.120 | 0.132 |
|  |  |  |  |  |  |  |  |  |  |  |  |  |  |  |  |  |  |  |
| Assemblage II | S Dellen | 0.172 | 0.115 | 0.178 | 0.220 | 0.204 | 0.112 | 0.103 |  |  | **-0.001** | 0.035 | 0.060 | 0.059 | 0.157 | 0.054 | 0.029 | 0.029 |
|  | N Dellen | 0.142 | 0.122 | 0.197 | 0.278 | 0.222 | 0.200 | 0.161 |  | 0.014 |  | 0.025 | 0.049 | 0.051 | 0.154 | 0.046 | 0.016 | 0.015 |
|  | Kalix | 0.173 | 0.128 | 0.191 | 0.263 | 0.227 | 0.171 | 0.154 |  | 0.002 | 0.008 |  | 0.044 | 0.053 | 0.179 | 0.062 | 0.019 | 0.014 |
|  | E Mälaren | 0.197 | 0.144 | 0.182 | 0.326 | 0.252 | 0.269 | 0.210 |  | 0.050 | 0.026 | 0.023 |  | **0.002** | 0.216 | 0.045 | 0.025 | 0.029 |
|  | W Mälaren | 0.323 | 0.227 | 0.224 | 0.408 | 0.340 | 0.387 | 0.358 |  | 0.126 | 0.096 | 0.096 | 0.019 |  | 0.209 | 0.038 | 0.030 | 0.031 |
|  | Rössjön | 0.477 | 0.254 | 0.170 | 0.370 | 0.334 | 0.427 | 0.472 |  | 0.249 | 0.284 | 0.281 | 0.207 | 0.211 |  | 0.211 | 0.163 | 0.173 |
|  | Siljan | 0.207 | 0.143 | 0.190 | 0.257 | 0.235 | 0.166 | 0.157 |  | 0.004 | 0.020 | 0.016 | 0.036 | 0.091 | 0.281 |  | 0.022 | **0.030** |
|  | Vänern | 0.168 | 0.132 | 0.196 | 0.297 | 0.241 | 0.218 | 0.181 |  | 0.021 | -0.004 | 0.003 | 0.013 | 0.072 | 0.278 | 0.016 |  | **0.004** |
|  | Vättern | 0.127 | 0.113 | 0.189 | 0.265 | 0.212 | 0.182 | 0.138 |  | 0.014 | -0.006 | -0.007 | 0.018 | 0.094 | 0.297 | 0.022 | -0.008 |  |
|  |  |  |  |  |  |  |  |  |  |  |  |  |  |  |  |  |  |  |


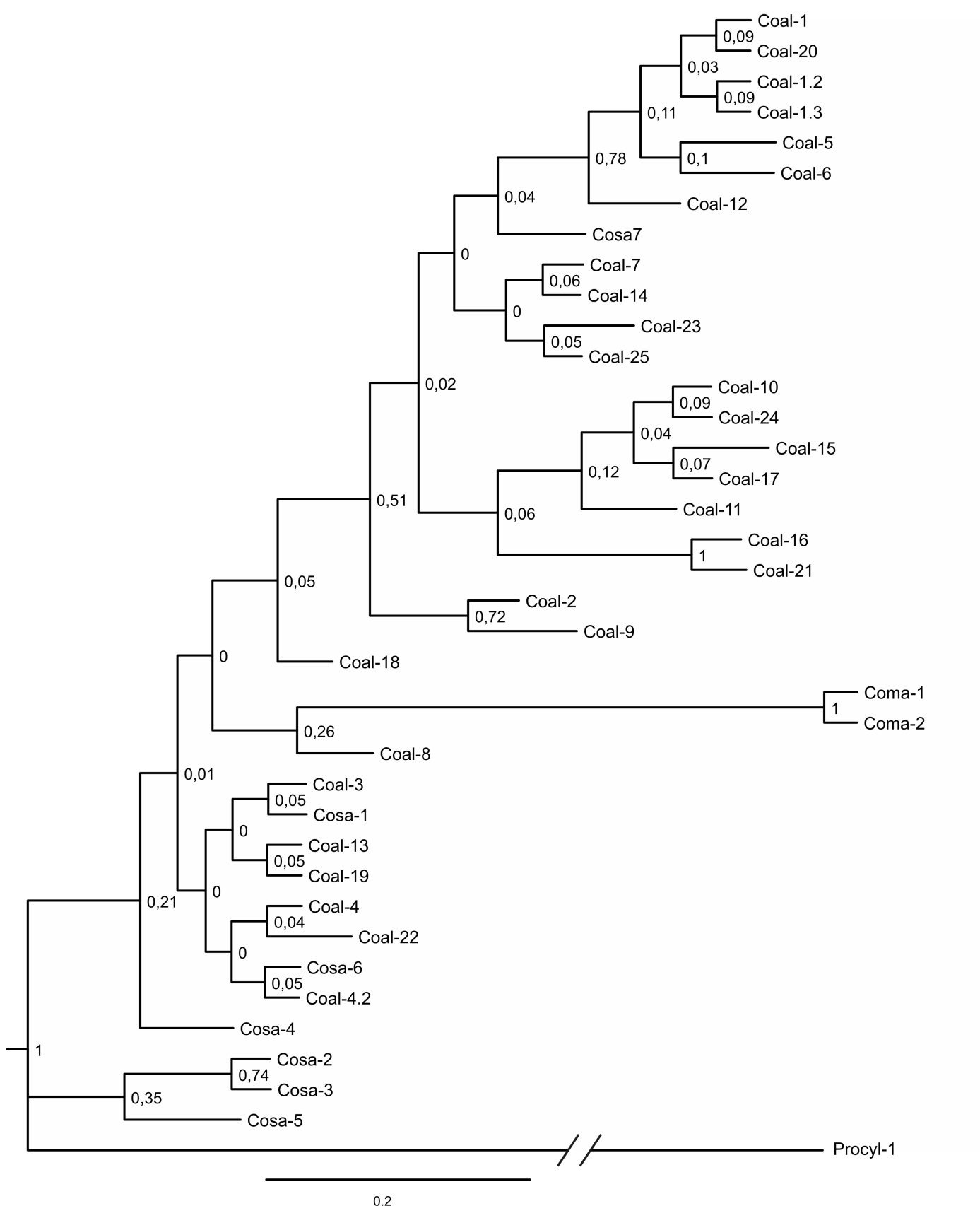


**Fig S1** Bayesian phylogeny estimated from the ND3 region of the mtDNA. Bayesian posterior probabilities are shown at the right of each node.


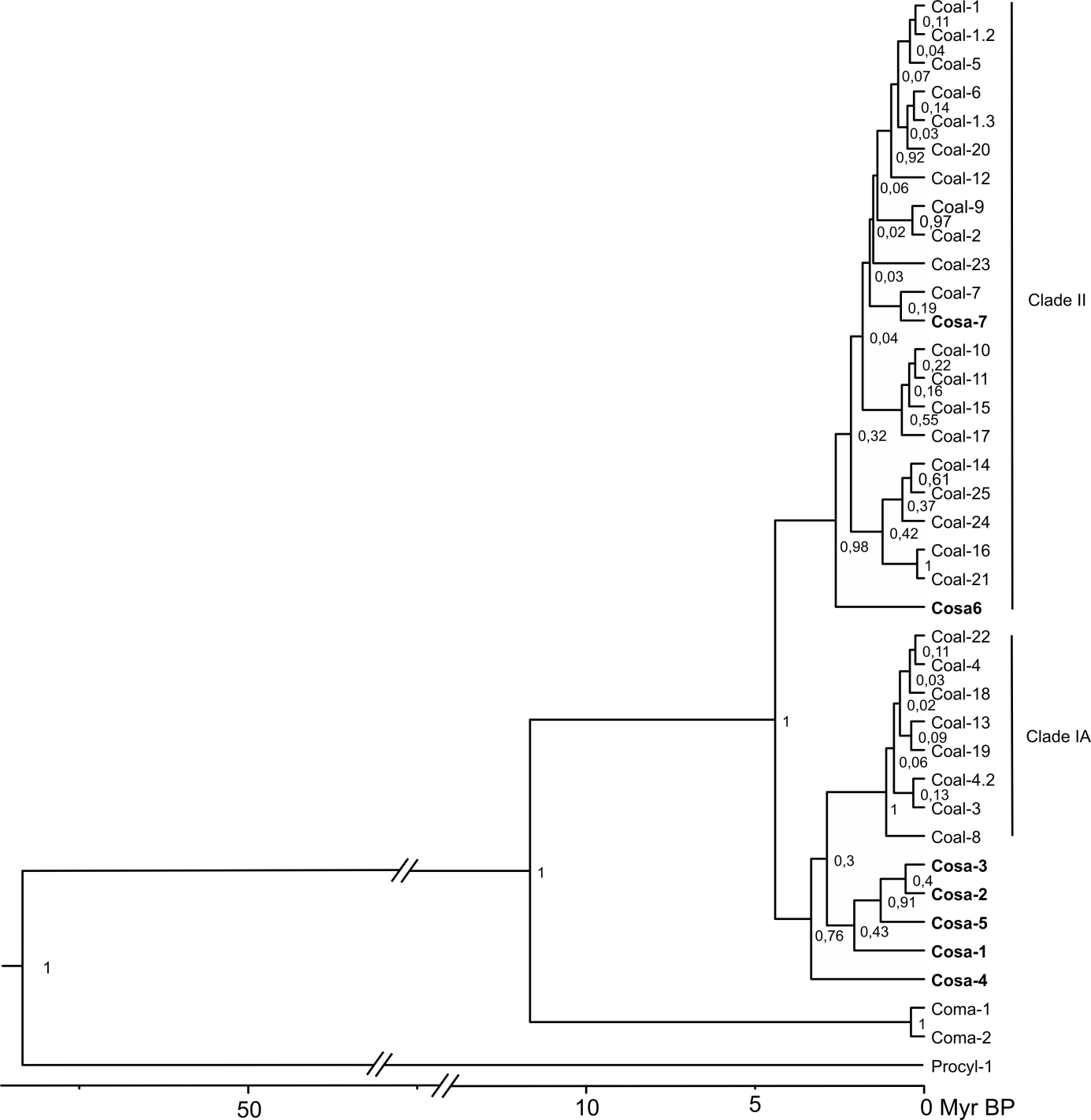


**Fig. S2** Dated Bayesian phylogeny estimated from the concatenated ND3 and D-loop region of the mtDNA. Bayesian posterior probabilities are shown at the right of each node.

**Fig. S3** Bubble diagrams (created with MS Excel) illustrating relative allele frequencies at nine microsatellite loci in Swedish ciscoes (samples listed in same order as in Table S5; i.e. population assemblage I at lanes 1-7; assemblage II at lanes 10-18). The rightmost lane (20) shows the corresponding allele frequencies in a population of *Coregonus maraena* (18 individuals from Lake Bolmen).

**Appendix S2**

*Estimation of population divergence time by Approximate Bayesian Computation (ABC)*

A simple **historical population model** including two populations originating from the same ancestral population was analyzed using microsatellite data and DIYABC 2.0 (Fig S4). Two separate analyses with the same basic model were performed. In the first analysis, time of divergence (t=t1) was estimated between the two distinct population assemblages within Sweden (present study). All individuals from present (t=0) populations belonging to populations within these assemblages were lumped into two large samples (Sa1 and Sa2; the aberrant sample from L. Rössjön was omitted). In the second analysis, we similarly estimated the time of divergence between the populations from the Kalix archipelago (Bothnian Bay) and L. Vänern. According to geological data, the two latter water bodies became isolated c. 9000 yBP, and we therefore wanted to check the inferred divergence time estimate (based on genetic data only) against prior knowledge based on independent information.

Flat uniform **prior distributions** were chosen for all modeled demographic parameters in both analyses (Table S7). Prior effective population sizes (N1-N3) were set to 10 – 500 000 diploid individuals, whereas the prior for the population split (t1) was set to 10 – 100 000 generations (i.e. 40 – 400 000 yBP, assuming a generation interval of 4 years). Initial test runs using a much higher upper prior limit for t1 (1 million generations) yielded poor congruence among simulated and observed data in principal component analyses and rank tests on summary statistics, using the pre-evaluation option available in DIYABC (not shown).

Priors for **mutational parameters** for the nine microsatellites were set as shown in Table S8, applying a generalized stepwise mutation model (GSM). A large difference in number of alleles per locus (range 4 – 51) indicated contrasting mutation rates at the markers studied. This observation, combined with poor initial congruence among simulated and observed data when using a common set of mutational parameters across all loci, prompted division of the nine loci into two locus groups that were given different prior mutational characteristics (a new feature in DIYABC ver. 2.0); we used default mutation priors for locus group 1 (three most polymorphic loci) whereas the prior mean mutation rate for locus group 2 (six less polymorphic loci) was reduced with one order of magnitude. The lower limit of the individual locus mutation rate prior in locus group 2 was also reduced accordingly (Table S8).

As one-sample **summary statistics** we used *mean number of alleles*, *mean genetic diversity*, and *mean allele size variance* across loci, whereas two sample summary statistics were *Fst*, *mean index of classification*, and *(dμ)^2^ distance*.

**Model checks** were performed using principal component analysis (PCA) of observed (real) and simulated summary statistics based on prior and posterior parameter distributions. Appropriate model fits was suggested by the PCAs as the observed data in each analysis was located within the cluster of simulated data sets based on the posterior predictive distribution (Fig. S5). Checking the two models against a separate set of five summary statistics not used for original estimation of the posterior parameter distributions resulted in similar goodness-of-fit (not shown).

Following simulation of 20 000 000 data sets, **posterior distributions** of demographic and mutational model parameters were estimated based on the 10 000 (0.5 ‰) simulations having summary statistics closest to those for the observed data set, as determined from weighted local linear regression on logit transformed parameters. Estimates of posterior parameter distributions (mean, median, mode, 95 % credibility interval) are listed in Table S9.

**Table S7** Priors for demographic model parameters (cf. Fig S4).

| Parameter | | Prior (uniform) | Explanation |
| --- | --- | --- | --- |
|  | |  |  |
| *Analysis 1 (divergence between two contemporary population groups)* | | | |
|  | N1 | 10 – 500 000 | N_e_ in Pop 1 (lumped populations within Assemblage I) |
|  | N2 | 10 – 500 000 | N_e_ in Pop 2 (lumped populations within Assemblage II) |
|  | N3 | 10 – 500 000 | N_e_ in ancestral population |
|  | t1 | 10 – 100 000 | Divergence time in generations (Assemblage I vs. II) |
|  |  |  |  |
| *Analysis 2 (divergence between local populations L Vänern and Kalix)* | | | |
|  | N1 | 10 – 500 000 | N_e_ in Pop 1 (Kalix population) |
|  | N2 | 10 – 500 000 | N_e_ in Pop 2 (L. Vänern population) |
|  | N3 | 10 – 500 000 | N_e_ in ancestral population |
|  | t1 | 10 – 100 000 | Divergence time in generations (L Vänern vs. Kalix) |

**Table S8** Priors for mutational model parameters (divided at two "locus groups"). Same parameters were used in Analysis 1 and 2.

| Parameter (distribution) | Locus group 1* | Locus group 2** |
| --- | --- | --- |
| Mean mutation rate (Uniform) | 1 × 10^-4^ – 1 × 10^-3^ | 1 × 10^-5^ – 1 × 10^-4^ |
| Individual locus mutation rate (Gamma, Shape=2) | 1 × 10^-5^ – 1 × 10^-2^ | 1 × 10^-6^ – 1 × 10^-2^ |
| Mean coefficient P (Uniform) | 1 × 10^-1^ – 3 × 10^-1^ | 1 × 10^-1^ – 3 × 10^-1^ |
| Individual locus coefficient P (Gamma, Shape=2) | 1 × 10^-2^ – 9 × 10^-1^ | 1 × 10^-2^ – 9 × 10^-1^ |
| Mean SNI rate (Log-u) | 1 × 10^-8^ – 1 × 10^-5^ | 1 × 10^-8^ – 1 × 10^-5^ |
| Individual locus SNI rate (Gamma, Shape=2) | 1 × 10^-9^ – 1 × 10^-4^ | 1 × 10^-9^ – 1 × 10^-4^ |

* *BWF1, BWF2, Sfo23;* ***Cisco90, Cisco126, Cisco157, Cocl23, Sfo8, Str73*

**Table S9** Estimated posterior parameter distributions based on 10 000 selected data sets (out of 20 000 000 simulated ones).

| Parameter | | Mean | Median | Mode | 95% cred. int. |
| --- | --- | --- | --- | --- | --- |
|  | | | | | |
| *Analysis 1 (divergence between two contemporary population groups)* | | | | | |
|  | N1 (Ne of population group I) | 167 000 | 143 000 | 117 000 | 34 300 – 426 000 |
|  | N2 (Ne of population group II) | 68 700 | 62 100 | 50 400 | 20 600 – 153 000 |
|  | N3 (Ne of ancestral population) | 50 500 | 40 200 | 22 800 | 2 360 – 156 000 |
|  |  |  |  |  |  |
|  | t1 (divergence time in generations) | 26 000 | 20 400 | 9 440 | 3 740 – 80 000 |
|  |  |  |  |  |  |
|  | Mean mutation rate (locus group 1) | 4.6 x 10^-4^ | 4.2 x 10^-4^ | 2.6 x 10^-4^ | 1.3 x 10^-4^ -- 9.4 x 10^-4^ |
|  | - " - (locus group 2) | 2.1 x 10^-5^ | 1.7 x 10^-5^ | 1.1 x 10^-5^ | 1.0 x 10^-5^ -- 5.8 x 10^-5^ |
|  | Mean coefficient P (locus group 1) | 2.3 x 10^-1^ | 2.4 x 10^-1^ | 3.0 x 10^-1^ | 1.2 x 10^-1^ -- 3.0 x 10^-1^ |
|  | - " - (locus group 2) | 2.3 x 10^-1^ | 2.4 x 10^-1^ | 3.0 x 10^-1^ | 1.2 x 10^-1^ -- 3.0 x 10^-1^ |
|  | Mean SNI rate (locus group 1) | 6.3 x 10^-7^ | 1.8 x 10^-7^ | 1.0 x 10^-8^ | 1.2 x 10^-8^ -- 4.5 x 10^-6^ |
|  | - " - (locus group 2) | 2.0 x 10^-7^ | 5.7 x 10^-8^ | 1.0 x 10^-8^ | 1.1 x 10^-8^ -- 1.3 x 10^-6^ |
|  |  |  |  |  |  |
| *Analysis 2 (divergence between local populations in L. Vänern and Kalix/N Baltic Sea)* | | | | | |
|  | N1 (Ne of Kalix) | 259 000 | 249 000 | 214 000 | 58 300 – 480 000 |
|  | N2 (Ne of Vänern) | 146 000 | 111 000 | 53 600 | 17 600 – 443 000 |
|  | N3 (Ne of ancestral population) | 61 500 | 51 100 | 24 500 | 3 890 – 176 000 |
|  |  |  |  |  |  |
|  | t1 (divergence time in generations) | 4 170 | 2 890 | 1 720 | 371 – 15 300 |
|  |  |  |  |  |  |
|  | Mean mutation rate (locus group 1) | 4.3 x 10^-4^ | 3.8 x 10^-4^ | 1.9 x 10^-4^ | 1.2 x 10^-4^ -- 9.4 x 10^-4^ |
|  | - " - (locus group 2) | 2.6 x 10^-5^ | 2.0 x 10^-5^ | 1.0 x 10^-5^ | 1.0 x 10^-5^ -- 7.7 x 10^-5^ |
|  | Mean coefficient P (locus group 1) | 2.1 x 10^-1^ | 2.1 x 10^-1^ | 2.8 x 10^-1^ | 1.1 x 10^-1^ -- 3.0 x 10^-1^ |
|  | - " - (locus group 2) | 2.3 x 10^-1^ | 2.4 x 10^-1^ | 3.0 x 10^-1^ | 1.2 x 10^-1^ -- 3.0 x 10^-1^ |
|  | Mean SNI rate (locus group 1) | 1.6 x 10^-6^ | 5.7 x 10^-7^ | 1.3 x 10^-8^ | 1.5 x 10^-8^ -- 8.1 x 10^-6^ |
|  | - " - (locus group 2) | 1.9 x 10^-7^ | 3.7 x 10^-8^ | 1.0 x 10^-8^ | 1.0 x 10^-8^ -- 1.6 x 10^-6^ |

**Figure S4** Simple demographic model used for two ABC-analyses. The model includes one divergence time parameter (t1) and three effective population sizes (N1-N3). Sa1 and Sa2 refer to samples taken from population 1 and 2, respectively.

**Figure S5** Principal component analyses (factors 1-3) based on summary statistics for real data (yellow dot) and simulated prior (green unfilled dots) and posterior (green filled dots) parameter distributions. Left panels: Analysis 1; Right panels: Analysis 2.

**References**

Drummond AJ, Ashton B, Buxton S, *et al*. (2012) Geneious v5.6. Available from *http://www.geneious.com.*

Filipsson O (1994) Nya fiskbestånd genom inplantering eller spridning av fisk. *Information från Sötvattenslaboratoriet*, (1994) **2**, 1-65.

Schulz M, Freyhof J, Saint-Laurent R, Østbye K, Mehner T, Bernatchez L (2006) Evidence for independent origin of two spring-spawning ciscoes (Salmoniformes: Coregonidae) in Germany. *Journal of Fish Biology*, **68**, 119-135.

Xia, X., Z. Xie, M. Salemi, L. Chen, Y. Wang. 2003. An index of substitution saturation and its application. Molecular Phylogenetics and Evolution 26:1-7.

Xia, X. and Lemey, P. 2009. Assessing substitution saturation with DAMBE. Pp. 615-630 in Philippe Lemey, Marco Salemi and Anne-Mieke Vandamme, eds. The Phylogenetic Handbook: A Practical Approach to DNA and Protein Phylogeny. 2nd edition Cambridge University Press.
